# Recent trends in nanocomposite packaging films utilising waste generated biopolymers: Industrial symbiosis and its implication in sustainability

**DOI:** 10.1049/nbt2.12122

**Published:** 2023-03-13

**Authors:** Zeba Tabassum, Anand Mohan, Narsimha Mamidi, Ajit Khosla, Anil Kumar, Pratima R. Solanki, Tabarak Malik, Madhuri Girdhar

**Affiliations:** ^1^ School of Bioengineering and Biosciences Lovely Professional University Phagwara Punjab India; ^2^ Department of Chemistry and Nanotechnology The School of Engineering and Science Tecnologico de Monterrey Monterrey Nuevo Leon Mexico; ^3^ Wisconsin Center for NanoBioSystmes University of Wisconsin Madison Wisconsin USA; ^4^ School of Advanced Materials and Nanotechnology Xidian University Xi'an China; ^5^ Gene Regulation Laboratory National Institute of Immunology New Delhi India; ^6^ Special Center for Nanoscience Jawaharlal Nehru University New Delhi India; ^7^ Department of Biomedical Sciences Institute of Health Jimma University Jimma Ethiopia

**Keywords:** food packaging, nanocomposites

## Abstract

Uncontrolled waste generation and management difficulties are causing chaos in the ecosystem. Although it is vital to ease environmental pressures, right now there is no such practical strategy available for the treatment or utilisation of waste material. Because the Earth's resources are limited, a long‐term, sustainable, and sensible solution is necessary. Currently waste material has drawn a lot of attention as a renewable resource. Utilisation of residual biomass leftovers appears as a green and sustainable approach to lessen the waste burden on Earth while meeting the demand for bio‐based goods. Several biopolymers are available from renewable waste sources that have the potential to be used in a variety of industries for a wide range of applications. Natural and synthetic biopolymers have significant advantages over petroleum‐based polymers in terms of cost‐effectiveness, environmental friendliness, and user‐friendliness. Using waste as a raw material through industrial symbiosis should be taken into account as one of the strategies to achieve more economic and environmental value through inter‐firm collaboration on the path to a near‐zero waste society. This review extensively explores the different biopolymers which can be extracted from several waste material sources and that further have potential applications in food packaging industries to enhance the shelf life of perishables. This review‐based study also provides key insights into the different strategies and techniques that have been developed recently to extract biopolymers from different waste byproducts and their feasibility in practical applications for the food packaging business.

## Graphical abstract

Utilisation of residual biomass could act as green and sustainable approach to lessen the waste burden on Earth and fulfiling the demand for bio‐based goods. Using waste as a raw material through industrial symbiosis should be taken into account as one of the strategies to achieve more economic and environmental value through inter‐firm collaboration on the path to a near‐zero waste society. This review extensively explores the different biopolymers which can be extracted from several waste material sources and can further be utilised for applications in food packaging industries to enhance the shelf life of perishables.

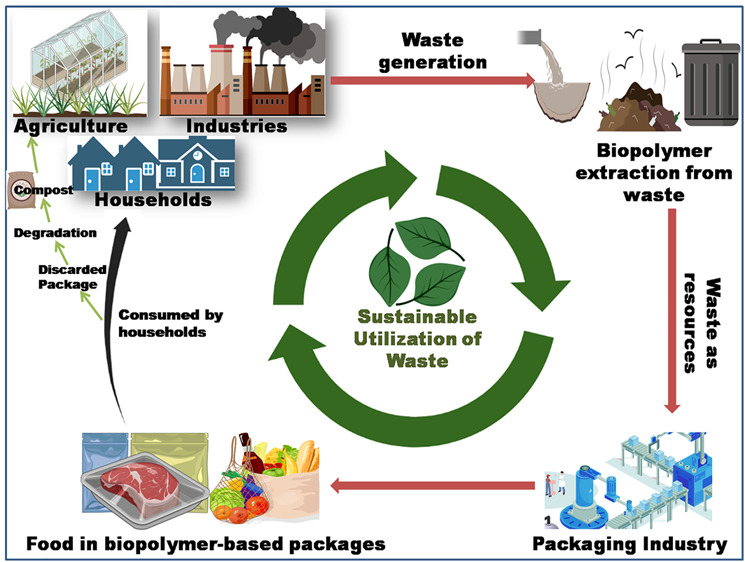

## 1 INTRODUCTION

The most urgent scientific, economic, social, and ethical challenge that humanity faces today is preserving our planet Earth as the ideal home for future generations. Unlimited waste generation and disposal challenges are causing irreversible damage to the environment as well as society and the economy. According to World Bank data of solid waste management, the annual trash generation is anticipated to rise by 73% from 2020 to 3.88 billion tons in 2050 due to increasing population expansion and urbanisation. For a rising nation like India, managing huge amounts of waste, including agricultural, municipal solid waste, and industrial hazardous waste, has become a crucial problem. The issue is concerning because only 12.45% of the waste is collected and technically managed, the remaining is dumped in open land fields [1]. The largest producer of food waste is the European Union, followed by India and the United States [2]. Evidence shows that greenhouse gas emissions from plastic waste occur at every stage of the plastic life cycle, including extraction and transportation of plastic raw materials, plastic manufacturing and waste treatment entering the environment [3, 4]. Although it is necessary to relieve this pressure on the environment, there is currently no effective solution for the treatment and reuse of waste residue processing. A long‐term, sustainable and smart solution is required, as the Earth's resources are limited. A large reserve of biomass in the form of waste is always unutilised or underutilised [5, 6]. Considering the increasing interest in the circular economy and bioeconomy, all waste should be recycled as profitably and sustainably as possible. This is especially important due to the severity of climate change, as well as the world's growing population and urbanisation. Recent concerns about the safe and sustainable disposal of solid waste have sparked interest in biotechnologies aimed at converting waste materials into bioenergy and biomaterials, thereby reducing economic reliance on fossil fuels. Utilisation of residual biomass residues emerges as a green and sustainable approach to address the waste disposal problem and meet the demand for bio‐based goods. Waste materials derived from agriculture, food processing plants and municipal organic waste can be used to produce biopolymers to valorise biomass at a reasonable cost. Biopolymers are a potential candidate to be applicable in various sectors, including the food packaging industry [7, 8] due to their biodegradability and compatibility [2, 9]. In agricultural and industrial processes, the biopolymer is one of the most common waste components. Finding new applications for these materials and repurposing biopolymer wastes into high‐value goods can encourage industrial symbiosis, a sustainable system. This article aims to present a review regarding different biopolymers which can be extracted from the waste and the extraction techniques and further application of those biopolymers in food packaging industries to prolong the shelf life of fresh goods when turned into bionanocomposites as an alternative to polymer‐based non‐degradable packages [10].

## 2 PLASTIC POLLUTION AND FINDING ALTERNATIVE

Over the last 6 decades, plastics have become an integral and essential part of our daily life, because of their versatility and wide range of properties including chemical composition and several applications in different fields. Plastics are superior to other conventional packaging materials (paper, wood, fabric, and metals) due to their availability, flexibility, durability, light weight, and economic viability. Despite the fact that plastic was once believed to be harmless and inert, many years of plastic disposal into the environment have resulted in a wide range of associated problems. Non‐degradable plastic waste pollution is now widely acknowledged as a major environmental burden [10]. From 1950 to 2018, approximately 6.3 billion tons of plastics were produced globally, among them only 9% was recycled and 12% was incinerated [11]. According to the International Energy Agency's World Energy Outlook, the largest application of plastic is in the packaging domain (26% of total volume) and is expected to grow steadily over the next years, with a potential of four‐fold increase by 2050, to approximately 318 million tons per year, which is more than the entire plastic industry today. Plastic packaging is one of the most problematic types of plastic waste because it is typically designed for single use, is common in the garbage, and is extremely difficult to recycle [3]. As of yet, environment‐friendly disposal platforms for synthetic polymers have not matured. As a result, their wastes tend to remain sources of pollution that emit hazardous substances regularly. 800 metric tons of polymeric waste has been reported to have been incinerated over the last 65 years (1950–2015). Although thermal treatment is the only way to fully degrade conventional polymeric wastes, incineration releases toxic pollutants such as dioxins, furans, Mercury, polychlorinated biphenyls, and others [12, 13]. The carbon footprint of plastic is around 6 kg CO_2_/kg plastic throughout the life cycle that is, production to incineration [14]. In its current form, greenhouse gas emissions from cradle to grave of plastics will reach 1.34 gigatons per year by 2030 and 2.8 gigatons per year by 2050 which will seriously consume the remaining global carbon budgets, thereby threatening the global temperatures to rise by 1.5°C–2°C by 2100. That is why the total global greenhouse gas emissions must be kept within the remaining carbon budget of 420–570 gigatons. If the plastic industry plans to expand its production on a large scale like this, then the problem will worsen further. The World Economic Forum forecasts for 2030, that the production and use of plastics will grow at an annual rate of 3.8%, and this growth rate will fall to 3.5% per year from 2030 to 2050 [3].

The production, import, storage, distribution, sale, and use of single‐use plastic have been banned in India from July 1, 2022. Bangladesh, China, Fiji, New Zealand, Namibia, Kenya, Portugal, Russia, and UK are the other countries to ban plastics. For all these reasons mentioned above, increased efforts have been made to develop biodegradable polymers that can be produced from a variety of natural feedstock to mitigate the aforementioned negative environmental impacts. Utilising a natural feedstock to produce biopolymers can be a desirable innovation if such products exhibit enhanced biodegradability while meeting technical specifications such as physicochemical properties and mechanical functionalities comparable to conventional plastics [4, 15]. Biopolymers synthesised from natural (plant/animal or microbial) sources are thus recognised as an appealing and alternative option to replace synthetic polymers in a variety of ways, as evidenced by their Life Cycle Assessment [10, 16].

## 3 BIOPOLYMERS: SUSTAINABLE GREEN MATERIAL

As a result of progress towards a more sustainable environment, green‐based materials are in high demand. One type of green asset that has full potential to largely replace synthetic polymers and their negative consequences is biopolymers and their composites. The use of biopolymers has enormous potential for natural ecosystem conservation and the prevention of further ecological destruction. According to the Intergovernmental Panel on Climate Change (IPCC), biopolymers could make a significant contribution to limiting global warming to 1.5 degrees Celsius by removing up to 20% of CO_2_ [17]. Recent research trends on natural biopolymers have shown positive growth in the case of long‐term global sustainable development strategies [18]. Biopolymers have received a lot of attention because of their unique properties, low cost, biodegradability, non‐toxicity, biocompatibility economic sustainability, ease of availability, and wide range of applications [19]. For example, Evonik, a German chemical company, recently developed different biodegradable polymer (medical) devices for long‐term drug‐release implants [20]. There is also a large body of work that supports biomedical applications of biopolymers for improved tissue engineering and drug delivery applications [21, 22, 23]. Furthermore, some microbes have been reported to naturally produce biopolymers that can be used efficiently in disposable items such as sanitary products and diapers [24]. Membranes made of biopolymers can also be used for gas separation, pervaporation, and water filtration [25]. Mainly polysaccharide and protein biopolymers come under the food category, which has been used inedible food coating [23]. Furthermore, recent research has been expanded to create biopolymer‐based sustainable and eco‐friendly edible or non‐edible food packaging [26, 27].

However, biopolymers tend to have lesser mechanical/chemical resistance compared to petroleum‐based plastics [19]. For practical application and to compete with plastic, efforts have been made by scientists to overcome such limitations. Natural plasticisers, plant extract or oil, nanoparticles, and others have been added [10, 28, 29, 30]. The use of polymer blends is also another promising alternative for improving biopolymer properties by limiting repulsive forces inside the biopolymer chains [31]. Thermoplastic starch and soy pulp from soy waste can form disposable items, packaging materials, and so on [32]. Oats and barely hull fraction utilised for paper making mostly met the same quality requirements as the commercial paper used for food packaging [33]. Even, unusual animal‐derived raw materials, such as feathers [34] and horns [35], are also used as polymer fillers by some researchers. To extend the shelf life of perishables, limiting oxygen movement is necessary. Due to increased molecular interaction and nanoparticle occlusion of pores, some bionanocomposite films can lower oxygen permeability rate, even better than low‐density polyethylene [36].

**FIGURE 1:**
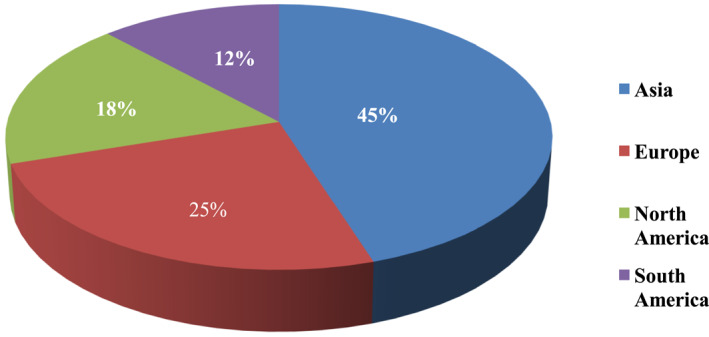
Bioplastic production around the world [41].

**FIGURE 2:**
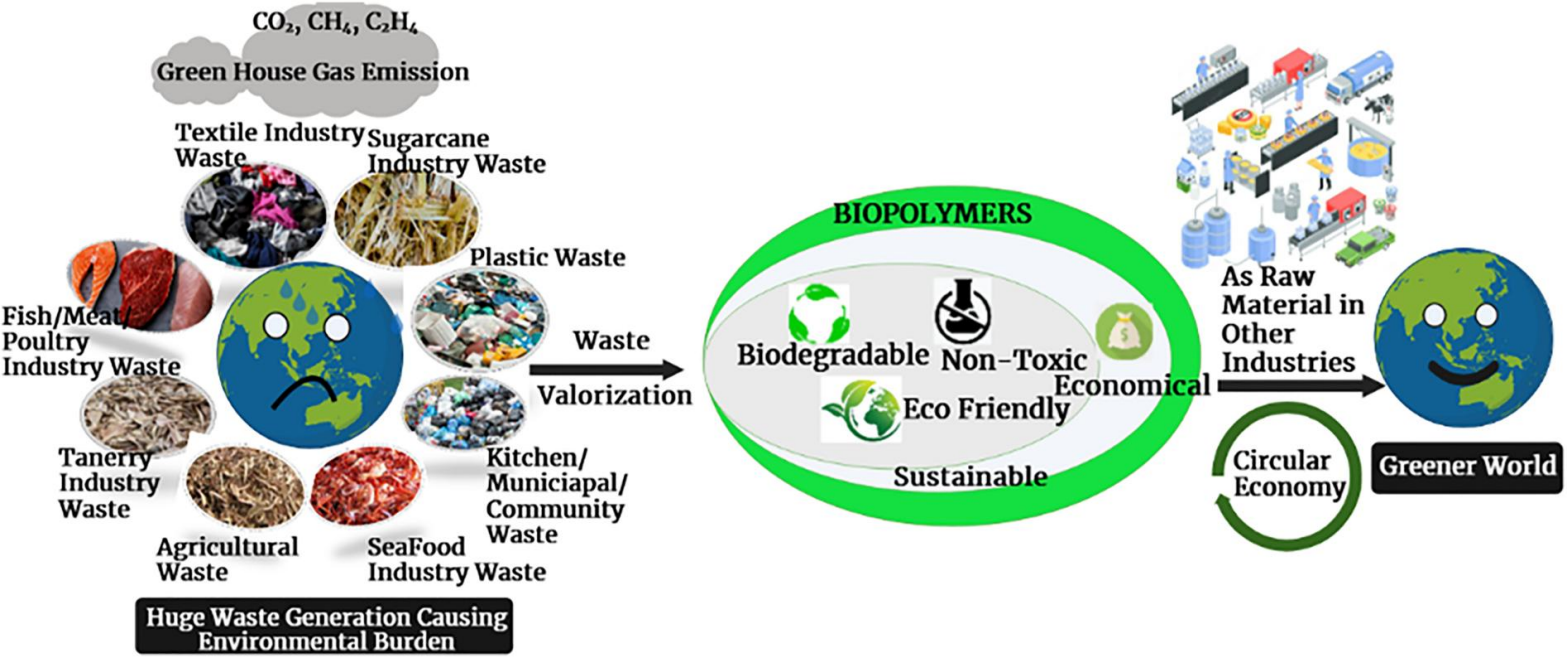
Role of biopolymers in a greener world.

Biopolymers derived from waste residues outperform other sources in terms of cost and sustainability [16]. Several researchers in this field claimed that biopolymers are overall better materials when compared to fuel‐based polymers, or other conventional materials [37, 38]. Annual publication on natural biopolymers was in its infancy from 2000 to 2002. There were a total of 67 articles published, and even though the number of articles at this stage was small, they were highly cited. The rapid growth of research in this area was observed with a total of 1694 publications from 2003 to 2017. It is in an explosive growth stage from 2018 to 2021, with a total of 1746 articles published, an average annual publication volume of around 437 articles, a total of 76,852 citations, and an average annual citation of around 19,213 times. The number of articles published each year is increasing with higher number of citations [18]. On the other hand, the biopolymer market size was 6.95 billion USD in 2018 and is expected to reach 14.92 billion USD by 2023, at a compound annual growth rate (CAGR) of 16.5% [39]. Even leading industries of modern services such as Samsung and Apple also have decided to use biopolymers in their products and packaging [40]. The European Union (EU) is the world's largest consumer of biopolymers, owing to stringent regulations and public demand for using biopolymers to mitigate the negative environmental impacts of synthetic polymers. The Asia‐Pacific biopolymer market is expected to grow steadily due to rising environmental concerns and global pressure to reduce pollution [40] (Figures 1, 2 and 1, 2).

## 4 BIOPOLYMERS FROM WASTE

Since the beginning of the Industrial Revolution, fossil fuels such as oil have been a critical energy source for virtually every commercial product, including the widely used plastic. However, because fossil fuels are limited resources, environmental concerns must be addressed when using them for both production and energy. We must act in a sustainable manner, which means using resources at a rate that allows them to be replenished by the natural cycles of our planet. Because of their renewable nature, biopolymers are experiencing a revival. Aside from the known sources of biopolymers, scientists all over the world are researching, developing, and even commercialising new products from a variety of renewable and waste materials.

Waste materials not only contribute to climate change but also have a significant impact on other environmental boundaries such as nitrogen and phosphorus cycles, global freshwater use, land composition change, chemical pollution, and biodiversity loss. Every kg of food protein waste can release up to 750 kg of CO_2_ into the atmosphere [42]. Different waste sources (industrial, agro‐industrial, agro‐food, and others.) contain biopolymers in the good amount [19, 34, 41, 43, 44, 45, 46, 47]. Using renewable resources for biopolymer production provides benefits such as reduced reliance on fossil fuels, waste management, resource recovery, and the creation of added value.

Agro waste containing fruits and vegetables are a rich source of starch [45]. Sugarcane industry waste contains lignocellulosic biomass, which is a reservoir of cellulose, hemicellulose, and lignin [48]. Cellulose can be obtained from agricultural waste in the huge amount [49], and even xylan and cutin can also be easily extracted from agricultural waste [50, 51]. Chitin/chitosan is the major byproducts of seafood industry waste [6], collagen and gelatin can be isolated from fish, poultry and tannery industry waste [8, 42]. The generated waste can be converted by microorganisms to produce polyhydroxyalkanoates [52], and xanthan gum [53]. All these biopolymers can be applied as raw material in other industries including the food packaging industry for the fabrication of eco‐friendly packaging [6, 19, 45, 54]. Being an interdisciplinary area of research that is a great blend of life science and engineering, it can offer new methodologies to redesign biosynthesis routes for synergistic behaviour in biomass conversion, eventually leading to economic and efficient strategies for transforming biomass into beneficial commodities such as biopolymers [55]. Kim et al. investigated the cost‐effectiveness and sustainable production of bioplastics from natural feedstock and found that organic wastes can significantly reduce costs [56]. The conversion of waste into useful packaging materials is now one of the top priorities for reducing the environmental impact of synthetic plastics. For example, the polymer produced by a strain of *Lactobacillus helveticus* in potato waste shows good thermal stability and a compact structure. Purified biopolymer exhibited strong antifungal properties against the fungal pathogen *Penicillium implicatum* [46]. The addition of ostrich eggshells to the levan biopolymer films reduced water vapour permeability while also demonstrating high antioxidant activity and antimicrobial action (against tested microorganism *Pseudomonas aeruginosa* inhibition was 68.6%) [57] (Table 1).

**TABLE 1:** Biopolymer extracted from waste source.

S.No.	Waste	Extracted biopolymer	References
1.	Agricultural waste for example, bagasse	Cellulose	[48, 58]
2.	The waste product of the starch industry (e.g. potato juice)	Bacterial cellulose	[59, 60]
3.	Textile waste	Nanocellulose	[61]
4.	Agro waste biomass (i.e. Banana leaf)	Cellulose nanofiber/nanocellulose	[62, 63, 64]
5.	Agro waste (e.g. Curaua fibres)	Hemicellulose	[65]
6.	Agro waste (such as fruit waste, and potato waste)	Starch	[66]
7.	Lotus stem waste	Starch	[67]
8.	Agro waste (potato peel, cassava, and corn residues)	Starch	[68]
9.	Plantain peel wastes	Starch	[69]
10.	Jackfruit seed	Starch	[70]
11.	Fish waste (*Cirrhinusmrigala* scale)	Collagen	[71]
12.	Fish waste (skin)	Collagen	[72]
13.	Fish waste (i.e. Scales of freshwater fish *Labeorohita*)	Gelatin	[73]
14.	Common carp waste (scales and fins)	Gelatin	[74]
15.	Chicken feet	Gelatin	[67, 75]
16.	Skin scrap	Gelatin	[76]
17.	Leather industry	Gelatin	[77]
18.	Eggshell waste membrane	Gelatin	[78]
19.	Chicken feathers waste	Keratin	[79, 80]
20.	Agro‐industrial waste fruit peel	Xanthan gum	[81]
21.	Potato crop residue	Xanthan gum	[82]
22.	Waste bread	Xanthan gum	[83]
23.	Kitchen waste	Xanthan gum	[84]
24.	Agro waste residue (such as corn, cob wheat straw, rice husks, maise stalks, and sugarcane bagasse)	Xylan	[50, 85, 86]
25.	Watermelon waste residue	Polyhydroxyalkanoate	[87]
26.	Poultry industry waste + Date pulp waste	Polylactic acid	[88]
27.	Agro waste (e.g. tomato waste)	Cutin	[51, 89, 90]

## 5 INDUSTRIAL SYMBIOSIS

The economic growth of emerging and frontier economies is facing several global challenges, one of which is related to the resources that are not reused in industrial activities [91]. As per the report ‘What a Waste 2.0: A Global Snapshot of Solid Waste Management to 2050’, waste production is getting higher every year and is expected to reach 3.4 billion tons per year by 2050 [92, 93]. Implementation of material reuse holistically can allow developing countries to grow industrially and socially while avoiding the risks of natural and non‐renewable resource depletion [94, 95]. Except for China and Brazil, there is a lack of understanding in emerging and frontier countries about the potential resources that can be recovered from industrial waste and the technologies required to reuse those [94]. Industrial symbiosis is the process through which wastes or byproducts from one industry or industrial process become raw resources for another [96]. Adopting this approach promotes more environmentally sustainable material use and aids in the development of a circular economy [97, 98]. Waste offers an amazing supply of biopolymers that are susceptible to transformation and valorisation, and such waste‐generated biopolymers also can be employed as raw materials for biodegradable food packaging material as a part of green technology [2]. For example, according to research, a low‐value biopolymer from agricultural waste that is, bagasse cellulose or an industrial by‐product generated biopolymer shrimp‐shell chitosan can be converted into packaging film with high‐performances [99, 100, 101]. These serve as the ideal illustration of an industrial symbiosis that promotes eco‐innovation. Furthermore, through industrial symbiosis, this waste can be processed for mutual economic and environmental benefit.

Industrial Symbiosis is intimately connected to industrial ecology, urban metabolism, process integration, and the circular economy. Research suggests that industrial symbiosis can reduce carbon emissions by 1.3% [95, 102]. Industrial symbiosis is an excellent way to dispose of and use solid waste, but developing countries face many challenges in solid waste management due to a lack of sustainable policies, lack of communication, trust among stakeholders, low use of technology in the industrial sector, and other technical barriers [94]. Costa and co‐workers did a case study in European countries on waste management policies for industrial symbiosis development and finally stated in their paper that coordination of policies and actions at the government level continues to be difficult. It is possible to contribute to more effective waste policies and shape the context for industrial symbiosis if there is enough flexibility to implement innovative, locally adaptable solutions and transfer their associated knowledge across levels [103].

## 6 LIGNOCELLULOSIC BIOMASS

**FIGURE 3:**
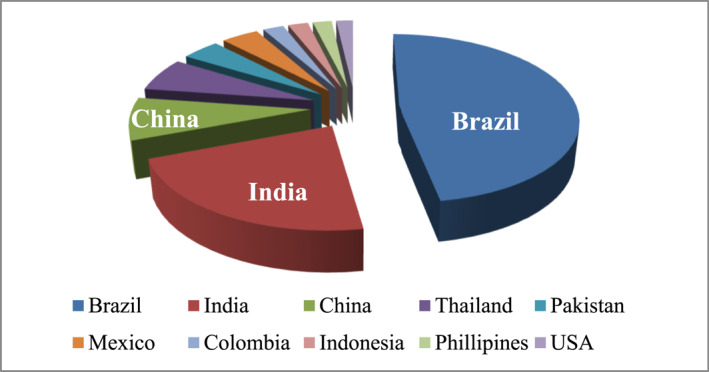
Highest production of lignocellulosic biomass worldwide: Brazil (739.3k TMT/year), followed by India (341.2k TMT/year) and China (125.5k TMT/year) [48].

Sugarcane bagasse is an inexpensive by‐product of the sugarcane industry and is a rich source of lignocellulose biomass, which is composed of structural components such as cellulose (crystalline region), hemicellulose, and lignin (amorphous region), as well as non‐structural components such as pectin and waxes [48, 104, 105, 106, 107]. The native form of lignocellulosic biomass is resistant to enzymatic degradation. It is because lignin is tightly cross‐linked with cellulose and hemicellulose which considerably inhibits enzyme engagement with cellulose and hemicellulose, decreasing sugar unit release. A variety of pretreatment procedures have been developed with the common goal of making biomass more sensitive to enzymatic saccharification. But it is the most expensive phase of conversion because it accounts for at least 20% of the total conversion's production costs [108]. There are several pretreatment techniques available, physical pretreatment (mechanical extrusion, milling, microwave, and others.), chemical pretreatment (acidic, alkaline, ionic liquid, and others) [109, 110], physiochemical pretreatment (liquid hot water, steam explosion, oxidative and others.), biological pretreatment (using microbial consortium, enzymes, and others) [111, 112, 113] (Figure 3).

**FIGURE 4:**
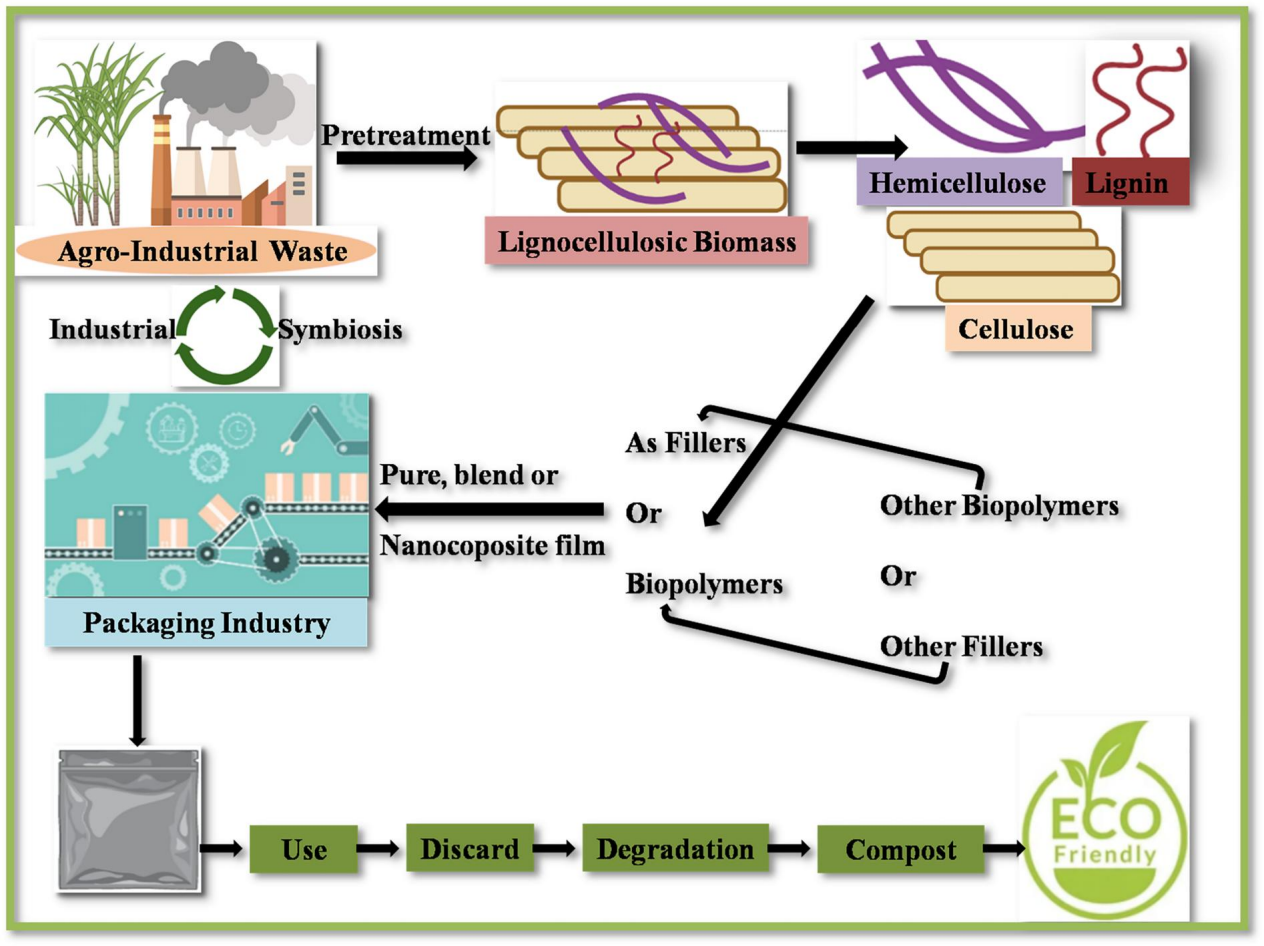
Lignocellulosic biomass valorisation and its implication in industrial symbiosis [121, 122, 123].

The most popular utilisation of lignocellulosic biomass is in the field of bioethanol production as an alternate source of energy [114, 115], other products that are generated from this biomass are biopolymers, xylitol, furfural, and many more [48]. Research proves that sugarcane pulp‐based materials can withstand temperatures of up to 200°F, making them suitable for use in an oven or microwave. This 100% natural material can easily degrade within a week and even acts as fertiliser while discarded [48]. Agro‐industrial waste offers a potential opportunity to gain economic benefits between agro‐industry and packaging businesses through industrial symbiosis. Other than cellulose, hemicellulose, and lignin‐based bio‐plastic, common biobased plastics that can be derived from lignocellulosic biomass include polylactic acid, polyhydroxyalkanoates [87], bio‐polyethylene, polyurethanes and other biopolymer‐based packaging incorporated with nanocellulose or lignin nanomaterial [9]. Research suggests that the thermal stability, antibacterial activity, and biodegradability of the package were improved significantly when lignin is blended with polylactic acid [116]. Montmorillonite nanoclay‐reinforced hemicellulose displayed higher tensile strength and elongation at break [117]. The blockage of pores into the polymer matrix by the nanofillers leads to decreased water and gas permeability and eventually enhances the barrier property of the film [36]. Furthermore, cellulose, hemicellulose, or lignin also has been used as a matrix in degradable packaging film applications [118, 119, 120] (Figure 4).

### 6.1 Cellulose

Agricultural waste for example, bagasse is a huge source of cellulose [48, 58], which is one of the most important materials utilised recently in the manufacturing of biodegradable or compostable polymers, including food packaging applications. This is mostly used as a filler or reinforcement agent in polymers to enhance their functional characteristics [124, 125]. Cellulose nanocomposites have a wide range of applications in pollutant removal, energy recovery, environmental sensing, and other sustainable commercial applications. Cellulose nanomaterials have many advantageous properties, including high mechanical and thermal stability, high specific surface area, and biodegradability [126]. One of the current food industry research trends is the use of bionanocomposites based on cellulose to meet food safety, economic, and quality requirements in packaging applications [127].

**FIGURE 5:**
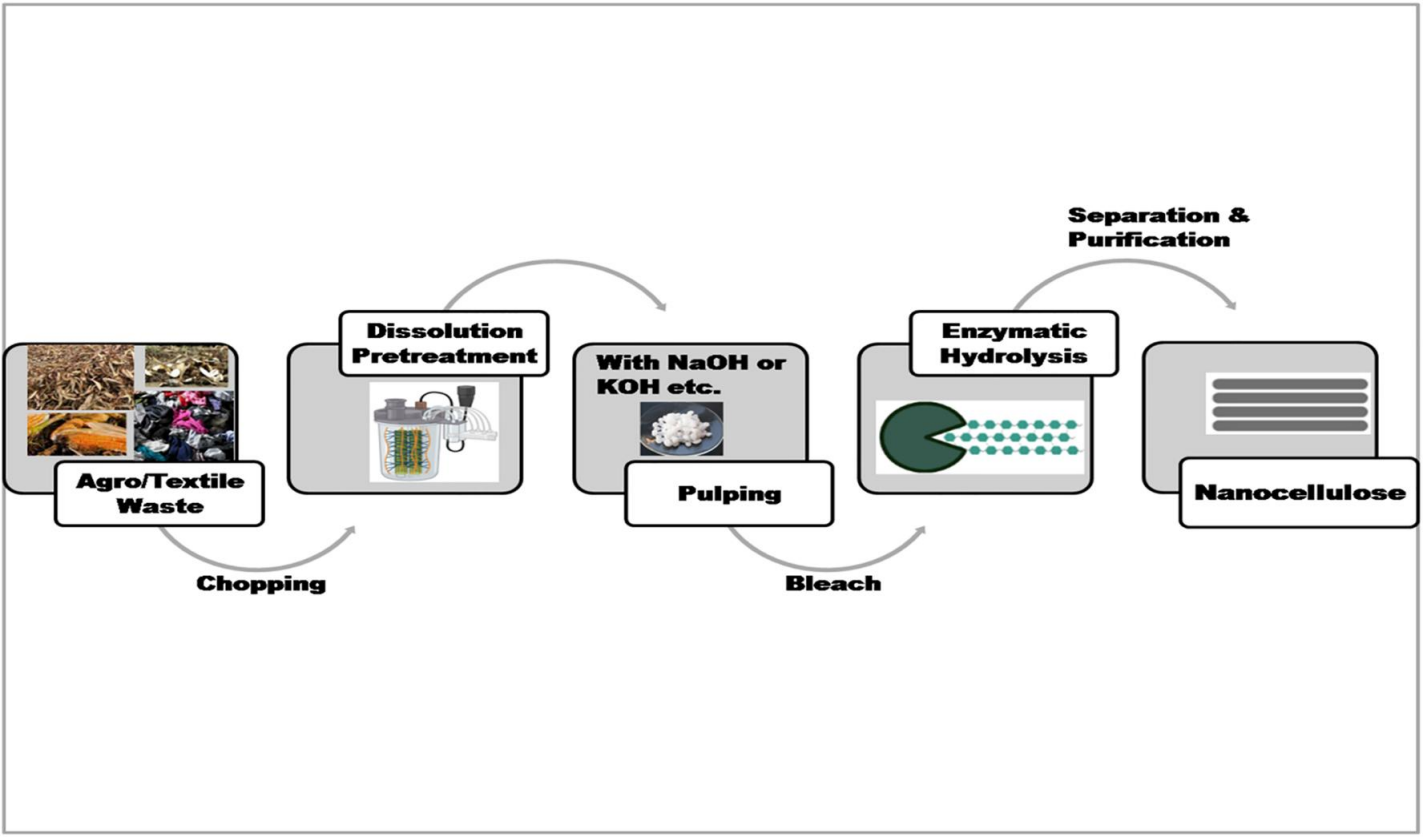
Nanocellulose production from agricultural/textile waste [61, 130, 131].

Cellulose Nanofiber was isolated from the waste banana peel through chemical treatment (Alkaline Treatment, Bleaching, and Acid Hydrolysis) to use as a reinforcing agent in Soy Protein Isolate films. The addition of these fillers didn't have any significant effect on its biodegradability, although strengthening in mechanical properties was observed [63]. With a reaction mixture of the xylanase enzyme at 70 U/g of banana peel bran, 15% substrate concentration, pH 6.0, temperature range of 35–55°C, this enzymatic hydrolysis proved very effective for cellulose nanofiber production. X‐ray diffraction revealed that the banana peel waste generated fillers presented a high crystallinity index of 66.2% [62]. Though there are other pretreatments (acidic, alkaline, and others.) available, enzymatic hydrolysis dismisses the need for solvents and chemicals, the mild conditions of this process make it economically attractive and environmentally friendly. However, the efficiency of enzymatic hydrolysis depends on factors, such as the hydrolysis time, the concentrations of substrate, enzyme, pH, and temperature. So, it is important to optimise the hydrolysis process to enhance its yield. Another nanocellulose fabricated from banana peel had shown strong and highly crystalline nature with high thermal stability and can play a significant role in the packaging of pharmaceuticals [128]. Fadeyibi et al. claimed that their fabricated starch‐banana peel nanocomposite film is a better packaging material than the conventional low‐density polyethylene since it recorded lower counts of the microbes throughout the storage of locust beans irrespective of the sample moisture and pH over the 30 days of packaging duration. Additionally, it has very low O_2_ (4%) and N_2_ (1%) gas permeabilities [129]. Textile waste is also a good source of cellulose for the development of nanocellulose. Olaiya et al. described an enzymatic‐assisted technique as a green approach that extracts nanocellulose from textile wastes [61] (Figure 5).

Bacterial cellulose is one of the purest forms of cellulose available from microbes. Scientists used potato juice for bacterial cellulose biosynthesis for creating a path towards significant valorisation of a waste product of the starch industry, but also towards a significant drop in bacterial cellulose production costs, enabling wider application of this biopolymer [59, 60]. This waste‐produced bacterial cellulose exhibited a similar antimicrobial effect against *Staphylococcus aureus* and *Pseudomonas aeruginosa* to the conventional medium. Even, the yield from the waste potato juice medium was greater than 4 g/L, despite the lack of any pre‐treatment [59]. Bandyopadhyay and coworkers fabricated bacterial cellulose‐based films combining them with polyvinylpyrrolidone and carboxymethyl cellulose. The film was transparent in nature and had good tensile strength, printability, and others [132]. Researchers utilised 70% v/v banana leaf extract and kombucha tea as fermentation medium to obtain bacterial nanocellulose and the yield achieved was 0.031 g nanocellulose/g of fermentation medium and the production rate was about 0.11 g L^−1^h^−1^ [64].

### 6.2 Hemicellulose

Hemicellulose is one of the main components in the lignocellulosic feedstock. Unlike cellulose, hemicelluloses consist of shorter chains, may have branches, and are of relatively low molecular weight, thus having low strength. To enhance its hydrophilic nature and low strength, researchers suggest some modifications [133]. Roldi‐Oliveira extracted hemicellulose through an alkaline method (using 10% *w*/*v* KOH, at 25°C for 3 h) from Curaua fibres and achieved a high yield. The film made of this hemicellulose had better thermal stability and elongation at break than other biodegradable polymers (PolylActic Acid and Poly hydroxybutyrate) and even polymeric films [65]. In another case, the alkaline (NaOH)‐alcoholic method was used to extract hemicellulose from sugarcane bagasse which was also used to prepare biodegradable package film. The increasing amount of hemicellulose can exhibit an improving trend in mechanical strength due to H‐bond formation between them [134]. Mango seed husk proved to be a good source of hemicellulose, with a high yield of 46.24%, when blended with xyloglucan and xylan, it formed a thermally stable film [135]. Hemicellulose‐pectin‐nanocellulose composite containing mango peel polyphenols showed good elongation of 12.99%, increased antioxidant activity (~22.5%), and has potential use as active packaging of fatty foods [136]. However, as the purification of hemicelluloses may not be economically feasible to upscale, sustainable and cost‐effective methods are needed to make their valorisation more realistic for industrial applications. Co‐components present in hemicellulose‐rich fractions may also provide added functionality, such as flavonoid content and antioxidant capacity [137].

### 6.3 Lignin

The natural occurrence of lignin was estimated to be between 0.5 and 3.6 billion tons, with an annual production of 100,000–200,000 tons from the cellulosic ethanol industry and 40–50 million tons from the pulp and paper industry [5]. It is worth noting that the lignin market will surpass USD 960 million by 2024, according to Ahuja and Deb from Global Market Insights [138]. Lignin is well‐known for its structural and support functions (mechanical properties) in plant cell walls and has great UV absorption capacity [139]. As a result, there has been a growing interest in research studies in recent years in applying lignin‐based materials (e.g. composites) as high‐performance and greener alternatives to fossil‐based polymers used in single‐use disposables, such as packaging films and membranes, towards potential plastic pollution solutions. Not only in lignocellulosic biomass from sugarcane is bagasse lignin also found in industrial waste as a byproduct of papermaking [140]. But conventional wastewater treatment processes are seldom efficacious for the complete removal of lignin from paper mill effluents. The biological process employing microorganisms (e.g. bacteria and fungi) for the degradation of lignin is regarded as an eco‐friendly, cost‐effective, and sustainable method. It is a one‐step treatment and/or amalgamation of various other physicochemical techniques [109]. Magalhaes and colleagues isolated lignin from pine sawdust using bio‐based and renewable chemicals, levulinic acid and formic acid together. The increase in temperature and extraction time (till 4 h) benefits the purity and yield of the lignin. However, applying only levulinic acid gave lignin with higher molecular weight due to their good affinity to each other [141]. The diverse functional groups in the lignin matrix make it a superior alternative for carbon‐based materials [140]. Lignin as filler can enhance the tensile strength of conventional cellulose paper from 40 to 400 MPa, and also improves the thermo stability and UV‐blocking performance [142]. It also proved to enhance elasticity and delayed the photo‐oxidative degradation of PolyButylene Adipate Terephthalate film [139]. Coconut coir pith is a relatively unexplored type of lignocellulosic waste from the coconut industry, significantly enriched lignin content (40.9 wt% as per the study of Lebedeva et al.). Lignin can be obtained with a 74% yield from coconut coir pith through the soda pulping technique [143].

## 7 COLLAGEN

Collagen is the most common animal protein because it is found in all connective tissues (such as skin, bones, ligaments, tendons, and cartilage) as well as interstitial tissues of parenchymal organs. However, as the triple helical structure of collagen prevents it from being broken down by enzymes, collagen is structurally the same strong molecule regardless of its sources [144]. Collagen is a widespread and important biopolymer in vertebrates, with a high degree of adaptability that allows it to be used in a wide range of applications in various fields. Because of its excellent biocompatibility, low antigenicity, and controlled biodegradability, collagen is a promising candidate for applications in the field of tissue engineering, cosmetic preparation, food coating or packaging and others. However, the high cost of commercial collagen limits its use. Chemical extraction of collagen often takes longer, so researchers are looking for a suitable process for extracting collagen from waste. The leather industry generates very problematic waste [145] and this tanning waste can be valorised to tailor‐made collagen biopolymers [146]. Collagen‐containing residues (e.g., bovine hides) are increasingly important sources from the meat industry that are often passed on to leather processing companies. Interestingly, the significant amounts of collagen present in such samples are not that well known. Marine waste collagen generally has a comparable or slightly lower molecular weight and a lower denaturation or melting temperature than mammalian collagen [147]. Various researchers have proposed suitable crosslinking treatments to improve thermal stability [148, 149]. The yield of fish byproduct waste collagen can reach more than 50% in dry mass, and the removal of oil during fish processing ensures the absence of any odour [144, 147]. The eggshell membrane, mainly composed of collagen‐like proteins, is readily available as a waste product of the egg industry [150].

Kulkarni and workers demineralised the *Cirrhinus mrigala* scales and extracted acid‐soluble collagen using acetic acid, with a yield of 2.7%. They also demonstrated that collagen from fish waste can be effectively valorised and used in the synthesis of novel biodegradable films with antimicrobial efficacy (against *Bacillus subtilis*, *Staphylococcus aureus*, *Escherichia coli*, and *Pseudomonas aeruginosa*) in conjunction with chitosan and neem extract [71]. Collagen when extracted from fish skin was used to make active food packaging films, while utilising carboxymethyl cellulose as a cross‐linker from *Berberis lyceum* root extract as an antioxidant. The films demonstrated outstanding characteristics such as high biodegradability, low transparency, high UV barrier properties, and excellent antioxidant activity. These films were also used to wrap mushrooms to extend their shelf life [72]. For being a highly conserved structural protein, collagen is an effective meat packaging material. The treated animal digestive tract (also known as the casing) is an excellent natural packaging wisdom for wrapping meat into sausage. It is the oldest and best edible meat packaging due to its thin walls, high toughness, and impact resistance. Different physical, chemical, and biological cross‐linking methods, as well as a variety of reinforcement approaches, including nanotechnology, are proliferating to improve the mechanical strength and barrier behaviours of collagen‐based packaging materials [151].

## 8 GELATIN

**FIGURE 6:**
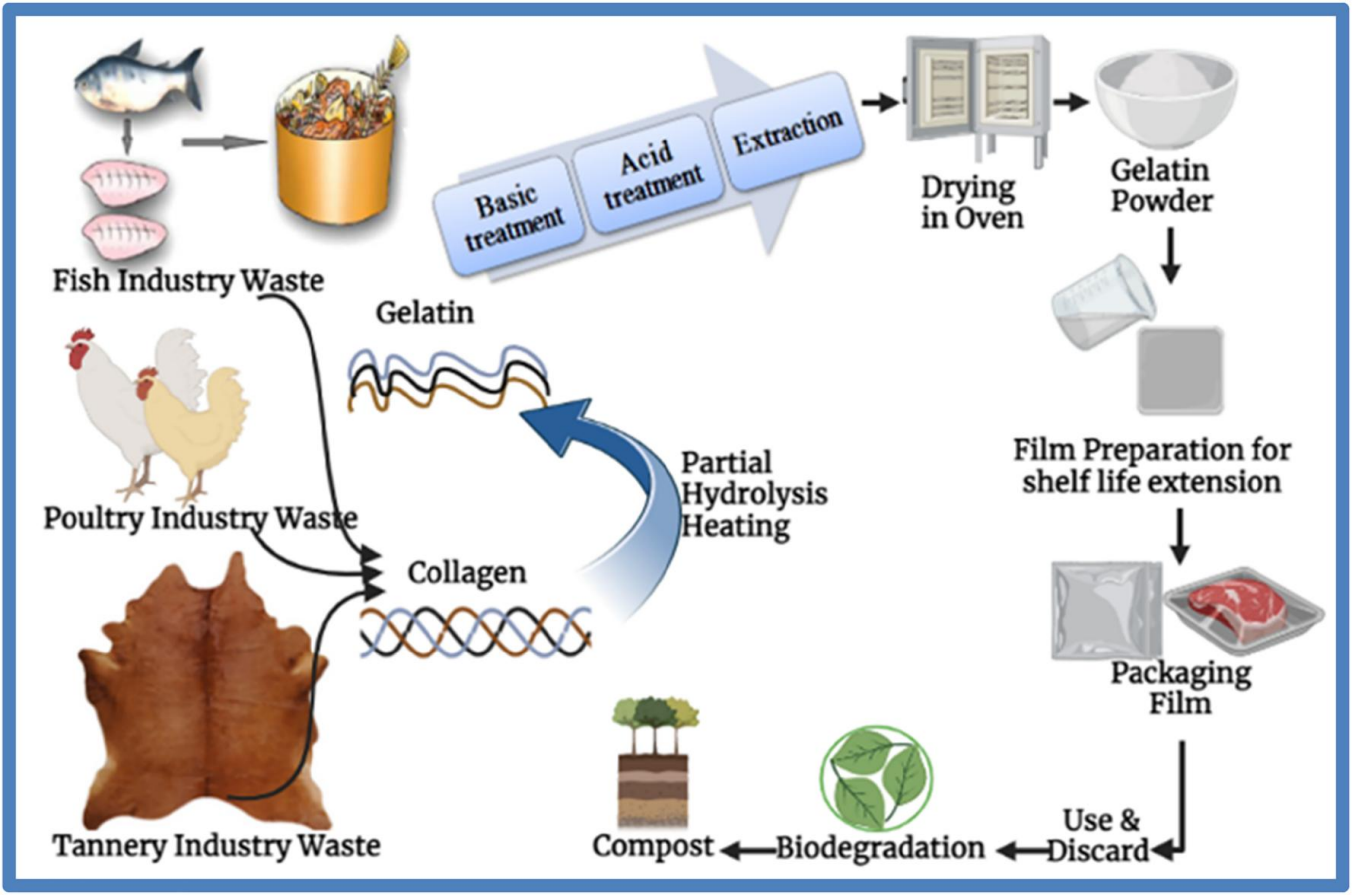
Gelatin from different sources, its extraction, and application in eco‐friendly packaging [75, 157].

According to the studies, global demand for all types of food and non‐food grade gelatins is expected to rise by 30% [152]. Gelatin is a biopolymer with excellent biocompatibility and biodegradability and is made from the thermal denaturalisation or partial hydrolysis of collagen. Gelatin has a wide range of applications in the food business [153]. Natural antioxidants and antimicrobials can be incorporated into gelatin‐based film‐forming formulations to extend food shelf‐life and reduce food waste [154]. According to Etxabide et al. active gelatin films derived from bovine, fish, and porcine gelatin can improve the quality of a variety of food products ranging from fish steaks to minced pork [154]. Ramos and coworkers concluded reviewing various related literature that food products coated with gelatin‐based films and coatings have been successfully utilised not only in the fish and meat industries, but also in fruits such as blueberries, pineapple, and strawberries, as well as some vegetables ranging from cherry tomatoes to carrots [155]. Costa et al. claimed that edible gelatin‐based coatings and films can improve the quality of a variety of cheese products ranging from cream cheese to cottage cheese [156] (Figure 6).

There is an increasing amount of research and published papers from 2010 to 2021 about the valorisation of fish industry waste (specifically fish waste gelatin) for biopolymer production and its application as food packaging [73, 74, 157]. Acid, alkali pretreatments, enzymatic extraction, ultrasound‐assisted extraction, and high‐pressure treatment are effective techniques to extract gelatin from fish waste [158].

Homogeneous, colourless, and transparent fish gelatin films demonstrate high resistance to UV light, which protects the packaged food product from oxidation reactions caused by light, allowing it to retain its quality for longer periods. It also proved to have a tensile strength of greater than 50 MPa and a positive environmental impact of composting it in its end‐of‐life scenario [159]. Gelatin extracted from fish wastes that is, scales of freshwater fish *Labeo rohita* also proved to have similar characteristics [73]. Kouhdasht and colleagues extracted alkaline protease from GRAS bacteria (*Bacillus licheniformis* PTCC1595) and utilised it to produce gelatin from common carp waste (scales and fins). The functional and physicochemical properties of commercial gelatin and fish waste gelatin, as well as their electrophoresis patterns, demonstrated that fish waste gelatin can be used as a good substitute for commercial gelatin [74].

Due to the increased consumption of poultry meat, the poultry industry sector has experienced spectacular growth in India since the late 1970s. South and West Indian states have led the way in this regard [160]. According to the website, The business research company, affirmed that ‘With a compound annual growth rate (CAGR) of 7.6%, the global poultry market will increase from $352.02 billion in 2022 to $378.84 billion in 2023’. A large amount of poultry waste is generated, which can cause environmental pollution if not properly disposed of, even though these wastes are high in protein and can be used as raw material in other industries for a variety of purposes [161]. Gelatin, a high molecular weight, and a hot water‐soluble protein with gel‐forming capacity are essential ingredients of poultry waste and byproducts obtained through partial collagen hydrolysis. Gelatin was extracted by Rather and coworkers from chicken feet through freeze‐drying and hot air drying and gelatin yields were 14.7% and 14.5% respectively. According to the researchers, such gelatin samples can be used to improve the functionality of various food products as well as to develop edible packaging materials. Gelatin from chicken feet and starch from lotus stem waste were recovered at 14.5% and 9.20%, respectively, and were used in different proportions to develop the coating to enhance the shelf life of tomatoes (Table 2) [67].

**TABLE 2:** Shelf life extension by biopolymer‐based packaging.

S. No.	Possible waste sources	Biopolymers/filler	Composites	Shelf life	Reference
1.	Fish, poultry, and meat industry waste	Gelatin as matrix	Active gelatin films	Improved the quality of a variety of food products ranging from fish steaks to minced pork	[154]
2.	Agricultural waste,	Starch,	Starch‐gelatin films	The packaging of marinated salmon significantly lowered total viable counts of microorganisms, which remained below the legal limit after 45 days of storage at 5°C. However, the water barrier seemed ineffective in preventing weight loss.	[162]
	Poultry, fish, or tannery industry waste	Gelatin			
3.	Cheese industry waste, sewage waste, agricultural waste, and others.	PolyHhydroxyButyrate	Polylacticacid‐Polyhydroxybutyrate	Enhanced shelf life by lowering the total bacterial count of salmon dice in chilled salmon storage	[163]
4.	Seafood industry waste	Chitosan as matrix	Chitosan reinforced with sodium montmorillonite and incorporated with ginger essential oil	Extending fresh poultry meat shelf life	[164]
5.	Agricultural waste	Sawdust particles as filler	Poly (lactic acid)/sawdust particle biocomposite film	Pangasius fish fillets inhibited the growth of Gram‐positive (Listeria monocytogenes, Staphylococcus aureus) and Gram‐negative bacteria (Pseudomonas aeruginosa, Aeromonas hydrophila, Escherichia coli, and Salmonella typhimurium) till 10 days	[165]
6.	Seafood industry waste	Chitosan as matrix	Chitosan‐cellulose acetate phthalate films incorporated with ZnO nanoparticles	Extended the shelf life of black grape fruits up to 9 days.	[36]
7.	Seafood industry waste	Chitosan as matrix	Mahua oil‐based polyurethane, chitosan incorporated with zinc oxide nanoparticles	Retard microbial growth and enhance the shelf life of cut carrot	[166]
				Pieces upto 9 days, more effectively than commonly used polyethylene bag	
8.	Agricultural waste	Starch	Starch‐glucose coating	Cucumbers' shelf‐life was extended upto 30 days with the least loss in weight, total soluble sugar, protein, catalase activity, peroxidase activity, and elevated proline content, 2, 2‐diphenyl‐1‐picrylhydrazyl antioxidant activities, and ferrous ion chelating activities	[167]
9.	Agro‐industrial waste, kitchen waste, and others.	Pullulan,	Gallic acid combined with epsilon‐polylysine hydrochloride incorporated in a pullulan‐CarboxyMethyl cellulose edible coating	Protein degradation, microbial growth, and lipid oxidation are all inhibited in stored sea bass (*Lateolabrax maculatus*) fillets stored at 4°C for 20 days	[168]
	Paper waste, waste of the cotton ginning industry, and others.	CarboxyMethyl cellulose			
10.	Paper waste, waste of the cotton ginning industry, and others.	Carboxymethyl cellulose,	Carboxymethyl Cellulose/Arabic Gum/Gelatin/Garlic Extract–TiO_2_ nanoparticles	During 21 days of storage of Nile Tilapia fish fillet at 4°C, microbial growth was controlled and weight loss decreased	[169]
	Poultry, meat, tannery waste	Gelatin			
11.	Agro waste,	Starch,	Cassava starch, chitosan, pineapple leaf fibre, and zinc oxide	For 30 days, the packaging tests with slice bread revealed antimicrobial properties with no fungal growth.	[170]
	Sea food industry waste	Chitosan			
12.	Waste tea ground	Carboxymethyl cellulose	Tea ground waste and furcellaran‐carboxymethylcellulose double‐layered films	Salmon	[171]
13.	Fish and meat industry waste,	Collagen,	Collagen‐carboxymethyl cellulose films enriched with Berberis lyceum root extract	Wrap mushrooms to extend their shelf life	[72]
	Paper waste, waste of the cotton ginning industry, and others.	Carboxymethyl cellulose			
14.	Agro‐industrial waste, kitchen waste	Xanthan‐gum	Xanthan‐gum‐based edible coating embedded with nano‐encapsulated *Litsea cubeba* essential oil	Potent antibacterial activity against *V. Parahaemolyticus which is responsible for the* deterioration of salmon quality at 4°C helped to extend the shelf life.	[172]
15.	Poultry waste,	Gelatin,	Gelatin‐starch film	Extend the shelf life of fresh cherry tomatoes, however, the firmness and ph of tomatoes were retained to a greater extent, and weight loss was minimised during the 15 days storage period	[67]
	Lotus stem waste	Starch as matrixes			
16.	Fish waste, eggshell waste, and others.	Collagen	Collagen/zein/gallic acid electrospun films containing 8% (w/w) gallic acid	Significantly halted the deterioration of tilapia muscle quality and extended the shelf life of fish muscle for at least 2 days.	[173]
17.	Agro‐industrial waste, kitchen waste	Xanthan gum	Hydroxypropyl methylcellulose film with xanthan gum	Banana shelf life has qualitatively improved, whereby the weight loss rate on bananas was able to decrease from 25 ± 3% (without coating) to 16 ± 4%	[174]
18.	Vanilla bean waste residue, PET plastic waste	Vanillin	Acrylic monomers‐vanillin film	High antimicrobial action helped increase the shelf life of a packaged meat product by 50%.	[175]
19.	Agro‐industrial waste, kitchen waste	Xanthan gum	Xanthan gum‐hydroxypropyl methylcellulose‐tea polyphenols composite film	A positive result was achieved, after 8 days, the amount of vitamin C retained was 127.81%.	[176]
20.	Agro‐waste	Starch	Polybutylene adipate‐co‐terephthalate and thermoplastic starch blended ZnO nanocomposite films	Able to extend the shelf‐life of packaged meat by more than 3 days in refrigerated condition	[177]
21.	Agro‐waste	Xylan	Nano ZnO@Xylan/quaternised Xylan/PolyVinylAlcohol film	Keep cherry tomatoes fresh for 21 days	[178]
22.	Agro‐waste	Starch, xyloglucan	Jackfruit seed starch/tamarind kernel xyloglucan/zinc oxide nanoparticles‐based composite films	When the prepared formulations were applied to tomato fruits, resulting in delayed quality loss and longer shelf life while showing effective antimicrobial action against *Staphylococcus aureus* and *Escherichia coli*.	[70]

Chen and colleagues extracted gelatin from waste skin scrap as an alternative option to plastic films. The film was created by introducing covalent bonds and metal‐ligand bonds into the gelatin matrix. The gelatin film displayed excellent mechanical performance with the highest fracture stress of 32 MPa, maximum fracture strain of 1.25 mm/mm, and a high Young's modulus of 471 MPa. It degrades in natural soil in approximately 50 days [76]. Gelatin is also one of the major components in solid waste in the leather industry, present in large quantities. When compared to gelatin extracted from animal bones, skin, and connective tissues, gelatin extracted from chromium‐tanned leather wastes displayed a lower molar mass due to the more aggressive extraction process required to disrupt chromium‐collagen bonds. As a result, chromium‐tanned leather wastes gelatin is more hydrophilic, making it more difficult to use in the production of polymers. To address this issue, films made with starch, chromium‐tanned leather wastes gelatin, and their blends were cross‐linked with glutaraldehyde. The cross‐linking reduced the crystallinity of the films, preventing gelatin chains from reorganising into a triple helix structure, which balanced the effect of the higher molecular chain while not affecting the film's tensile strength. It increased elongation at the break while decreasing solubility and swelling by up to 53% and 69% respectively. The authors stated in their findings that this study represents a significant step forward in the practical application of starch‐chromium‐tanned leather wastes gelatin films [77].

The addition of chitosan to eggshell waste membrane gelatin resulted in increased elongation at break significantly, effective enough to apply in flexible packaging. Water solubility and vapour permeability decreased due to the great interaction between the biopolymer matrixes and the formation of homogenous and compact networks [78]. A similar finding was observed in chitosan‐gelatin‐nano ZnO film with additional 94.92% improved crystallinity and higher antibacterial properties [179].

## 9 KERATIN

Like other wastes, the valorisation of keratinous wastes increases the waste value and enables more sustainable waste management towards a circular bioeconomy. The abundance of keratinous wastes as feedstock from the agro‐industrial processing, wool processing, and grooming industries can be beneficial in terms of keratin extraction, which may be the best solution for developing an environmentally and economically sustainable keratin‐based economy. The transition from the current harmful process that ends energy and resource recovery through incineration and landfilling to the environment‐friendly model, a more sustainable and closed‐loop recycling and recovery approach that will minimise pollution, disposal challenges, and loss of valuable bioresources [180].

**FIGURE 7:**
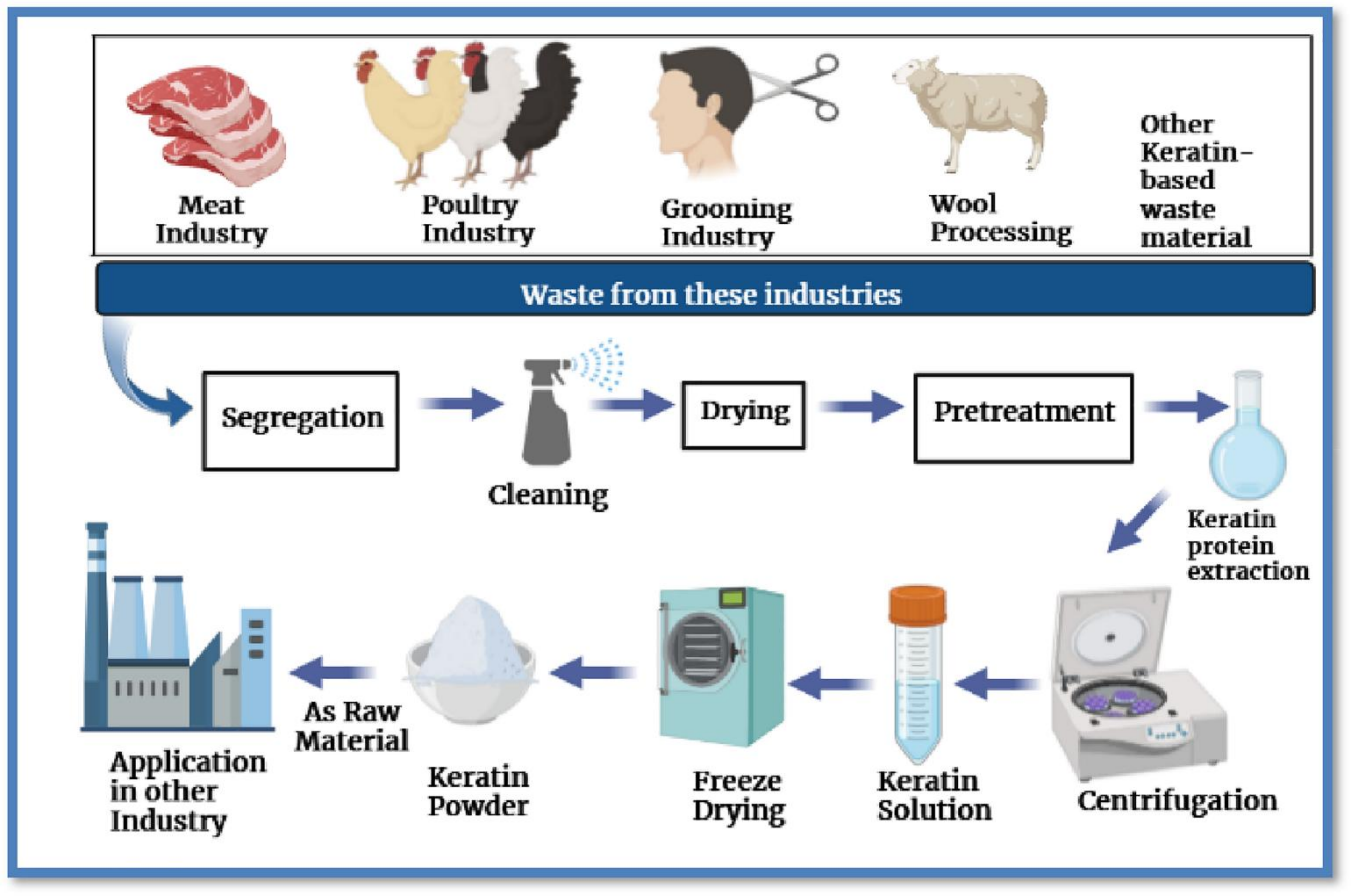
Steps of keratin extraction from different waste sources [186, 187].

The FAO (Food and Agriculture Organisation) estimated global chicken meat production of 128 million tons per year, accounting for 40% of total global meat production in 2020 [181]. Increased production has resulted in an increase in waste such as litter, feathers, eggshells, carcasses, blood, and wastewater. Each year, 66 billion chickens [182] weighing 134 million tons are processed globally. This generates 68 billion tons of waste per year globally [183]. Poultry industry waste containing keratin protein and several amino acids can be used to prepare biopolymers [34, 88]. Waste poultry feathers are an abundant source of renewable and sustainable keratin‐derivable material (composed of more than 90% keratin protein). The global poultry industry is estimated to generate 8–9 million Mt of waste bird feathers per year. And, with the sector currently growing at a compound annual growth rate (CAGR) of 3.8% and is expected to reach a CAGR of 7% by 2025 [184, 185] (Figure 7).

Other sources of keratin include the leather and textile industries, animal wool, nail, horns and hoofs, human hair, and so on [186, 188]. In recent years, significant efforts have been made to harness the potential of keratin and derived materials for the production of films, composites, hydrogels, and other materials for sustainable development [189, 190]. Nonetheless, one limitation of obtaining keratin from waste poultry feathers is the use of harsh chemical processes that are not environmentally friendly. Additionally, the molecular weight of the derived keratin is low, limiting its optimisation [185]. As a result, the scientific community must devise environmentally friendly methods to efficiently derive keratin materials with high molecular weight from waste poultry feathers for a variety of applications.

Çelik et al. 2021 developed keratin‐chitosan biofilm by extracting keratin from waste chicken feathers, the highest keratin extraction value were obtained after 6 h at 60°C and 400 rpm, with 0.5 M Na_2_S per 5 g of chicken feather powder. This is one of the promising methods that have great potential for the industrialisation of keratin bio‐waste due to its enormous extraction performance using common and inexpensive chemicals and simple equipment under ambient conditions [79]. Oluba et al. combined keratin from waste chicken feathers with ginger starch to create a biodegradable composite film that displayed increased transparency indicating better UV‐barrier properties, an appealing quality for food packaging materials. Keratin was found to improve the toughness of ginger starch films by increasing tensile strength and elongation at break, possibly due to intermolecular interactions between the starch's functional groups of starch and keratin. Furthermore, the moisture content that leads to microbial attacks on the fabricated biofilms was found to decrease significantly, indicating the potential for the improved shelf life of packaged products [80]. As a potential replacement for fossil‐based plastic packaging, edible dialdehyde carboxymethyl cellulose cross‐linked with feather keratin and plasticised with glycerol was cast into a packaging film. The produced film, which is completely biodegradable and environmentally friendly, demonstrated good UV‐barrier properties (due to the aromatic/amino functional groups) and transmittance. As previously stated, in terms of food safety regulation, the higher the UV‐barrier, the better for food packaging because oxidation and discolouration of packaged food items are undesirable. However, despite the low cost, environmental friendliness, transiency, and potential for food packaging applications of the films, the moisture content stability of the films were irregular due to the presence of hydrophilic groups inherent in the DCMC [191].

## 10 STARCH

Starch is a polymeric carbohydrate, synthesised by most green plants composed of many glucose units linked together by glycosidic linkages. Because of the abundance of their raw materials and the ease of processing with existing methods, starch‐based biopolymers have stimulated the interest of many researchers. Rice, bread, and potatoes are widely consumed and account for a significant portion of global food waste, making them a potential feedstock for starch production [192]. In both developing and developed nations, the expansion of the potato industry has pushed it to one of the top spots, the fourth most abundant crop harvested annually worldwide [193, 194]. Agro‐industrial fruit wastes are also promising sources of biopolymers like starch [66, 195]. The global industrial starch market size was valued at USD 97.85 billion in 2020 and is expected to expand at a compound annual growth rate (CAGR) of 7.0% from 2020 to 2028 [196].

Starch extraction from plantain peel wastes has demonstrated waste utilisation and the production of value‐added goods. The average starch yield (from dry mass) was 29% in this case, while purity reached over 70% [69]. Starch has several advantages such as being cheap, plentiful, renewable, biodegradable, and starch‐based bioplastics can be processed using standard techniques such as solution casting, extrusion, injection, compression moulding, and others [197]. Some properties of starch‐based bioplastics, however, must be improved to compete with petroleum‐based plastics. Starch, in particular, has low water resistance as well as poor mechanical and thermal properties. Some of these properties can be enhanced by using filler materials. Lignocellulosic waste materials are among the most used intriguing fillers in starch‐based bionanocomposite films. As lignocellulosic materials are polysaccharides with chemical structures similar to starch, there is a good interaction possibility between them. The use of lignocellulosic waste materials may increase the value of the waste while decreasing the volume of landfill. The waste fibre from banana inflorescence produces effective powder filler that can improve the performance of starch‐based bioplastic. The optimal filler content for maximum tensile strength and minimum mass loss in water was 10% by weight. SEM analysis revealed good adhesion between the filler and the starch matrix, and the hydrogen bonding between the filler particles and starch chains was confirmed using Fourier transform infrared spectroscopy [198].

Lignin‐containing cellulose nanofibrils fabricated by mechanically fibrillating unbleached tree bark after alkaline extraction and used as reinforcement in Zhang and colleagues' biodegradable composite thermoplastic starch film. When 15 wt% lignin‐containing cellulose nanofibrils were added to the composites, the tensile strength and modulus had a huge increase of 319% and 800%, respectively, when compared to neat thermoplastic starch films. The mechanical properties of the composite films were improved by the crystalline property of cellulose and the high interaction between thermoplastic starch and lignin‐containing cellulose nanofibril [199].

Pineapple shell, which is considered waste in the juice industry, was used as a reinforcement material by Nakthong et al. developed a compression moulding process to produce biodegradable foam trays based on cassava starch. All trays were semi crystalline, with the 95/5 ratio having the highest crystallinity index value (39%). Pineapple stems are also part of agricultural waste in pineapple‐producing countries because pineapples must be replanted every 2 years. Pineapple stem starch, which was extracted mechanically wet‐grinding, is a resistant and thermoplastic starch for food packaging [195].

Agricultural by‐products such as jackfruit seed and tamarind kernel were utilised to extract starch and xyloglucan respectively and further used to develop biodegradable food packaging by reinforcing with zinc oxide nanoparticles. The film got reduced hydrophilicity, while increased material strength, improved UV and water vapour resistance. The film's increased glass transition and melting temperatures indicated improved thermal stability. The XRD results show that the addition of ZnO nanoparticles increased the crystallinity of the nanocomposites, which is an important parameter because crystallinity inhibits the pathway of oxygen molecules and also helped to increase the strength [70, 200]. Tesfaye and colleagues concluded in their study that producing industrial‐grade starch from waste avocado seeds is technically and economically feasible, with an accounting rate of return of 75% and a break‐even analysis of 82% over a 2 years payback period. Their described process is environmentally sustainable because it uses minimal chemicals in the extraction process and the wastes generated can be beneficiated into high‐value product formations that can increase the venture's profitability [201]. This extraction process requires the use of 0.3% (w/v) sodium metabisulphite for 3 h at 40°C, resulting in a 56% industrial‐grade starch yield. A financial analysis of the project demonstrates the technology's economic feasibility, with a payback period of 2 years and a return on investment of 75.12. Selling the industrial‐grade avocado seed starch for $0.8375/kg demonstrates that the project is financially viable [201]. Green biofilms were also prepared by combining starch and keratin extracted from waste avocado seeds and waste chicken feathers respectively [187].

Not only as a biopolymer matrix, but scientists also developed pathways to create non‐toxic starch nanoparticles to be used as fillers in other composite films and able to alter the film's physicochemical, functional, and mechanical properties such as low solubility, low water vapour transfer rate, making them a potential material for food packaging applications [202].

## 11 CHITIN/CHITOSAN

Modern seafood processing techniques generate a significant volume of waste products, which frequently include several high‐value products that remain untapped because of lacking appropriate management. This seafood waste frequently contains a high concentration of chitin, a polysaccharide with exceptional inherent properties such as biocompatibility, biodegradability, antimicrobial, and antioxidant activities. Chitin is the most abundant polysaccharide in the marine ecosystem and the second most abundant in nature after cellulose and has enormous potential to meet consumer needs in a variety of commercial sectors. Natural chitin is produced about 100 billion tons from different sources each year [203]. Living organisms in the ocean produce approximately 10^12^–10^14^ tons of chitin/year [204], among them, arthropods produce 2.8 × 10^10^ kg in freshwater and 1.3 × 10^12^ kg in the marine environment [204, 205]. Chitin Market size was valued at $42.29 Billion in 2020 and is projected to reach $69.297 Billion in 2028, growing at a CAGR of 5.07% from 2021 to 2028 [206]. Because of its versatility in applications ranging from biomedicines to textiles, food, pharmaceuticals, and cosmetics, chitin has proven to be a profitable way to convert waste from the marine food processing industry into valuable products. Even though chitin has the potential to provide outstanding commercial properties, its industrial application in various sectors is still limited due to issues with optimising mechanical and biological properties according to the intended application. As a result, intensive research is required for the design and optimisation of chitin processing methods into various forms and potential reinforced materials for controlling properties within a desirable range.

**FIGURE 8:**
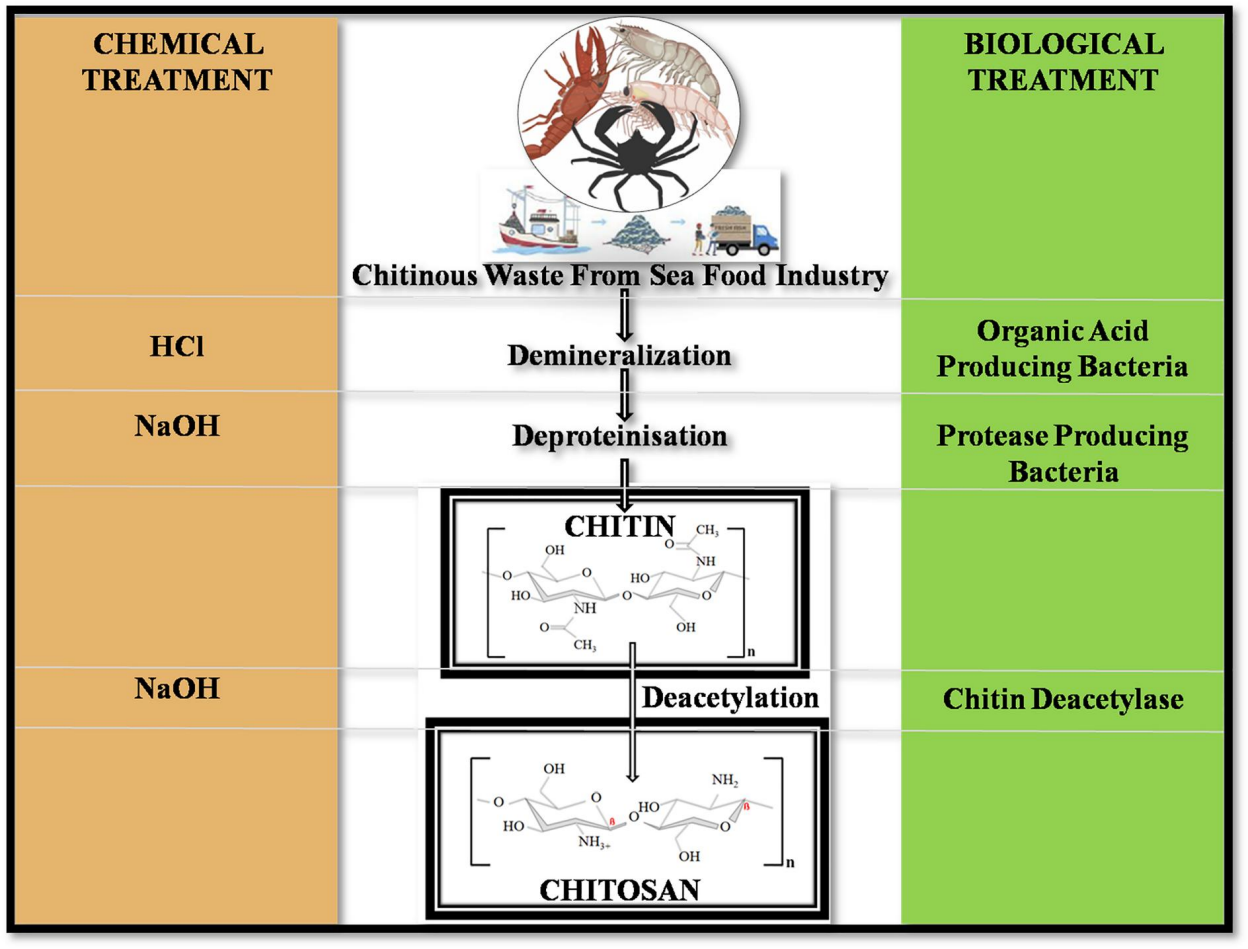
Production of chitin and chitosan from marine industry byproduct [207].

Chitosan is produced from the deacetylation of chitin, as shown in Figure 8. (Figure 8). Chitosan is a cationic biopolymer (C_56_H_103_N_9_O_39_) that contains deacetylated β‐(1–4) D‐glucosamine and acetylated N‐acetyl‐D‐glucosamine that is randomly distributed. Charles Rouget, 1859 was the first scientist to synthesise chitosan through the deacetylation of chitin [208]. The degree of deacetylation can affect both chemical and biological properties (tensile strength, solubility, surface area, viscosity, conductivity, porosity, and flexibility) (adsorption enhancer, biodegradability, antioxidant, bioavailability, and biocompatibility).

The use of harsh chemicals in extraction methods, on the other hand, frequently results in the production of polymers with variable properties. As a result, greener and cleaner processes, such as biological extractions are preferable, while minimising the amount of irregular by‐products. Pérez and colleagues recently developed an eco‐friendly process for chitosan production from waste shrimp shells. Demineralisation with citric acid is followed by enzymatic deproteinisation with papain or bromelain to produce chitin, followed by deacetylation to produce chitosan. A high yield of 1 kg chitosan/kg shrimp shell was achieved with 64% deacetylated, 87.5% deproteinised, and 98% demineralised [209].

The natural film‐forming ability of chitosan and its inherent antimicrobial action makes it suitable for food packaging. A hypothesis proposed by several scientists demonstrated that the positively charged amino group of chitosan interacts with the negatively charged cell wall of microorganisms, altering the membrane structure and causing intercellular component leakage, ultimately leading to cell damage and death, was observed by the researchers when the pH of the surrounding environment is acidic [210]. Another theory holds that chitosan binding to microbial enzymes blocks the active center, slowing microbial growth [211]. Chitosan (low molecular weight) has the potential to penetrate microorganism cell walls and bind to DNA, halting mRNA and protein synthesis and leading to cell death [212]. Chitosan with a high molecular weight can form an impermeable layer on the microbial cell, preventing it from absorbing nutrition [212, 213]. Furthermore, chitosan chelates the environmental ions and nutrients required for survival and may inhibit mitochondrial ATP production [214].

Tomato Byproduct Extract in PolyVinylAlcohol and chitosan film conferred significant antimicrobial effects on the tested microorganisms (against *S. aureus* and *P. aeruginosa*) [215].

Carotenoid pigments extracted from the fruit wastes (*Bactris gasipaes*) using ethanolic and aqueous solutions of ionic liquids and surfactants. The maximum extraction yield of carotenoids was 88.7 ± 0.9 μg carotenoids ⋅ g dried biomass^−1^. Carotenoids were successfully used in the preparation of chitosan‐based films to incorporate functional activity for an alternative food‐packaging material, with excellent results in terms of mechanical parameters and antioxidant activity [216].

## 12 XANTHAN GUM

Xanthan gum is a branched heteropolysaccharide biopolymer, comprising pentasaccharide units (d‐glucose, d‐mannose, and d‐glucuronic acid) residues in the molar ratio of 2:2:1. (C_35_H_49_O_29_)n was discovered by Allene Rosalind Jeanes and her research team in the early 1960s [217]. This is a very popular component in recent times, generally used as a food additive, stabiliser, and thickener [217]. The Xanthan gum market is expected to grow at a rate of 4.5% between 2019 and 2024 and will reach a valuation of 1.2 billion USD [218]. However, Xanthan gum production from synthetic media is costly, therefore, several attempts have been made to produce xanthan gum from agro‐based and food industry wastes. This valuable component can be extracted from different waste sources such as waste bread [83], agro waste residual peel (waste) of *Mangifera indica* (Mango) and *Manilkara zapota* (Sapodilla) using *Xathomonas* [81], kitchen waste [84], and others. Prasun and his colleagues gave an effort to produce crude xanthan gum in a cost‐effective manner using substrate like residual peel (waste) of *Mangifera indica* (Mango) and *Manilkara zapota* (Sapodilla) by the organism *Xanthomonas campestris* followed by fermentation, optimisation at pH 6.0 for mango peel and pH 7.4 for Sapodilla peel. The product yield achieved was 20.84 g/L and 11.83 g/L for Mango and Sapodilla media respectively, using chilled isopropanol. Xanthan gum obtained from these wastes was compared to 4.8 g/L from standard media containing yeast, glucose, malt, and peptone [81].

Xanthan gum yield of 14.3 g/L was obtained by *Xanthomonas axonopodis vesicatoria* when waste bread was used to produce xanthan gum. The highest conversion rate of waste bread to xanthan gum was found as 14.1% for *Xanthomonas hortorum* pv. *pelargonii*. Whereas, the highest aqueous solution viscosity of gum produced from standard bacteria was 11.2 Pa. s^n^ at a glucose ratio of 4%, inoculum volume of 5%, and mixing rate of 225 rpm. [83]. Li P and colleagues developed a cost‐effective solution for the reusing of kitchen waste and a possible low‐cost approach for xanthan production. Kitchen waste was firstly pretreated and fermented and the maximum yield reach up to 11.73 g/L. the thermal stability of the xanthan gum obtained in this study was similar to the commercial sample, claimed by the researchers [84]. Xanthan gum from untreated potato crop residue 6.2 g xanthan gum/100g substrate. After the pretreatment at the optimum conditions, the highest xanthan gum amount was achieved 12.5 g/100 g substrate [82].

The addition of xanthan gum to other biopolymers proved to enhance tensile strength and elongation at break [219]. The antioxidant and antibacterial activity was also increased, demonstrating the good inhibitory ability of *Staphylococcus aureus* [176].

## 13 XYLAN

Xylan can be extracted from a variety of agro‐waste residues, such as wheat straw, rice husks, maize stalks, sugarcane bagasse, which are abundant but currently underutilised, and others [50]. Xylan is the most common form of hemicellulose which is a class of heteropolymers consisting of pentose and hexose sugars. Xylan films exhibit properties that are suitable for food packaging applications such as the formation of transparent films, low oxygen permeability, and strong barrier to oils and fats [220]. Naidu and Jhon claimed that nanoclay‐reinforced xylan‐based nanocomposite film can protect food from lipid oxidation. However, the introduction of nanomaterials seemed important here to improve mechanical (tensile strength and Young's modulus) and barrier properties [85, 178].

## 14 VANILLIN

Vanillin is a bio‐based polymer, the primary component of the extract of the vanilla bean, used for flavour and aroma in food, beverages, and pharma. The market demand for vanillin was higher than 15,000 tons annually and the global market demand for vanillin reached 37 tons in 2018 [221, 222]. But the vanillin production from vanilla pods is too expensive and insufficient. Due to the high consumer demand for natural or organic food production, many efforts were made to produce natural vanillin through the biotransformation process. As per the studies of Syafrizayanti et al., five *Bacillus* sp. bacteria (*Bacillus cereus, Bacillus thuringiensis*, *Bacillus thuringiensis*, *Bacillus cereus,* and *Bacillus cereus*) were obtained from the Spent Bleaching Earth waste are capable of transforming isoeugenol to vanillin [222]. In another case, the chemical extraction of ferulic acid from agro‐industrial waste raw coir pith ensured bioconversion into vanillin. Ferulic acid, a precursor of vanillin, was extracted from the raw coir pith by chemical pre‐treatment and used as a substrate for biotransformation by *Asper*g*illus ni*g*er* to vanillic acid, which, in turn, was fermented by using strain *Phanerochaete chrysosporium* to vanillin [223].

Commercial acrylic monomers with covalently linked vanillin motifs possessed antibacterial activity for *Escherichia coli*, *Staphylococcus aureus*, and *Listeria monocytogenes* with an inhibition percentage of up to 99.96%. And above all, this packaging film is easily washable and reusable for at least 10 cycles [175].

Polyethylene terephthalate is an abundant and extremely useful material, with widespread applications across society. However, there is an urgent need to develop technologies to valorise post‐consumer polyethylene terephthalate waste to tackle plastic pollution and move towards a circular economy. Sadler and Wallace recently reported a novel pathway in engineered *Escherichia coli* for the direct upcycling of post‐consumer PET bottle‐derived monomer terephthalic acid into the value‐added small molecule vanillin. The reaction is mild, uses a whole‐cell catalyst produced from renewable feedstocks occurs under ambient conditions (room temperature, pH 5.5–7), in aqueous media, requires no additional cofactors or reagents, and generates no hazardous waste. Maximum vanillin titres of 785 μM (119 mg L^−1^, 79% conversion) were achieved [224]. This is one of the very innovative studies and can be very helpful for a greener world if it can be implicated practically in a real‐world situation.

Recently there are some emerging studies done by some scientists to use vanillin in bio‐packaging to enhance tensile strength and decrease water vapour and oxygen permeability [225, 226].

## 15 SHELF LIFE EXTENSION

**FIGURE 9:**
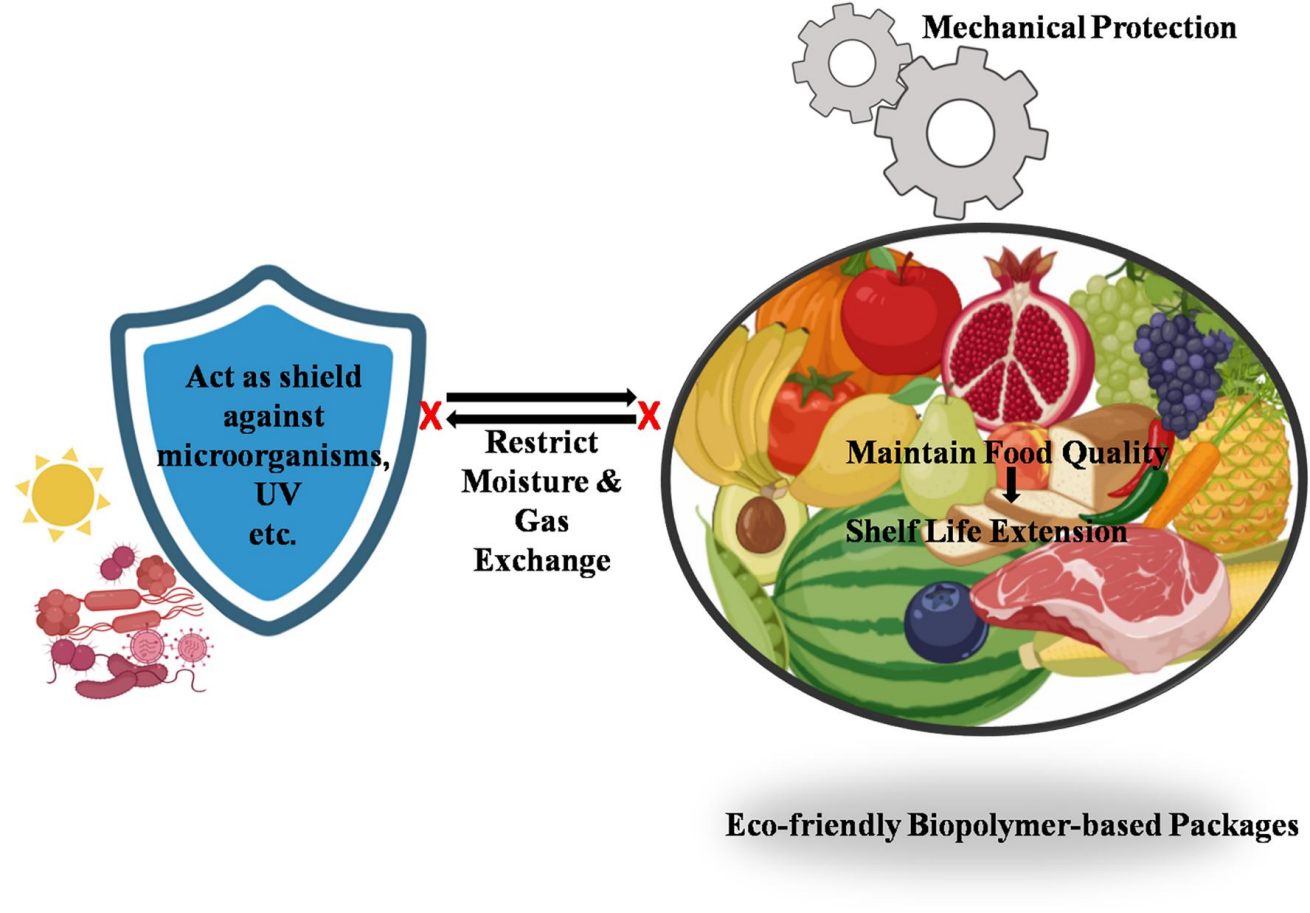
Shelf life extension by biopolymer‐based packaging [26, 227, 228].

As the world's population is expected to exceed 10 billion by 2050, there is an urgent need to increase food supplies to meet the demands of future generations. Reduced food losses during the post‐harvest stage are critical to ensure global food security. Fresh produce comes under perishable commodity due to their biological nature. Enzymatic and microbiological deterioration severely reduces the shelf life of these products at room temperature or even in refrigerator storage conditions. Therefore, the ideal postharvest practices are required to maintain product quality for a sufficient amount of storage time. Due to the enormous environmental pollution caused by the disposables used in our modern civilisation, biodegradable packaging materials made from renewable resources and natural polymers are required when considering postharvest practices. The nanotechnology field has gained a lot of interest from the researchers to develop ways for the preservation and value addition of food products, particularly perishable goods, while also improving their quality and safety along with shelf life. Recent bio‐based innovative edible/non‐edible and active films are a promising sustainable preservation technology for extending the shelf‐life of perishable food products by inhibiting decay kinetics and limiting the mass transfer of moisture, aroma, or gases. Antimicrobials, antioxidants, light blockers, and barriers are some of the components found in active packaging materials [161]. (Figure 9) (Table 2).

By 2024, the global market for smart packaging is expected to reach $26.7 billion [229]. Smart packaging refers to packaging systems that include embedded sensor technology and is used with foods, pharmaceuticals, and a variety of other products. It's used to increase shelf life, monitor freshness, display quality information, and improve product and customer safety. Furthermore, smart packaging provides new business opportunities based on digitisation and thus falls under the purview of Industry 4.0 [229]. Food manufacturers are increasingly interested in developing smart and active biodegradable packaging materials to improve the sustainability and environmental impact of their products while maintaining their quality and safety. Smart packaging materials contain sensing components that detect changes in food attributes, such as changes in quality, maturity, or safety. A smart sensor, for example, may produce a measurable colour change in response to a decrease in food quality [230] (Table 3).

**TABLE 3:** Biopolymer‐based smart packaging.

S. No.	Possible waste sources	Biopolymers/fillers	Composites	Indicator of shelf life	References
1.	Tannery, poultry, or fish industry waste,	Gelatin,	Edible intelligent packaging of gelatin, gellan gum, and red radish anthocyanins extract	Able to present visible colour changes in the presence of milk and fish spoilage	[231]
	Biodiesel derived waste	Gellan gum			
2.	Agro‐industrial waste (e.g. banana peel)	Nanocellulose	Intelligent pH‐sensing indicator based on bacterial nanocellulose and black carrot anthocyanins	Showed distinguishable colour changes during fresh (deep carmine colour), best to eat (charm pink colour), and spoiled (jelly bean blue and khaki colours) stages of both fish fillets (rainbow trout and common carp)	[232]
3.	Waste corncob,	Xylan,	Polyvinylalcohol/chitosan/xylan/hydroxyapatite‐based biodegradable hybrid nanocomposite	The colourimetric behaviour of PCC can be considered and used as a tool to assess the real‐time fish freshness method for smart packaging.	[233]
	Conch shell	Chitosan			
4.	Paper waste, cotton ginning industry waste, agro‐waste	Carboxymethyl‐cellulose/starch	Carboxymethyl‐cellulose/starch and purple sweet potato (Ipomoea batatas[L.] Lam) anthocyanins	The intelligent film is an indicator of NH3 and pH changes, so can be used to monitor the real‐time freshness of fish	[142]
5.	Agro waste (e.g. jackfruit seed)	Starch	Smart film from jackfruit seeds containing anthocyanin extract	Freshness indicator of fish	[234]
6.	Biodiesel‐derived waste	Gellan gum	Active and intelligent gellan gum‐based packaging film incorporating Clitoriaternatea	Colourchanging properties help to monitor shrimp spoilage	[235]
7.	Seafood industry waste and cellulose or lignocellulose based agro‐waste	Chitosan as a matrix,	Polylactic acid‐coated nanocellulose/chitosan‐based film	Potential to monitor spoilage in beef and other meat products refrigerated at 4°C	[236]
		Nanocellulose as filler			
8.	Marine‐derived waste	Chitosan as base matrix	Chitosan and broken Riceberry phenolic extract Marine‐derived waste	Produced intense naked‐eye detectable colourimetric response of freshness in seafood products., when enclosed with fresh shrimp, CHI‐RPE film changed from orange‐red to yellow in response to shrimp spoilage	[237]

## 16 BIODEGRADABILITY

To reduce the negative environmental impact of solid waste, demand for biodegradable packaging materials to replace synthetic plastic is growing. Biodegradable packaging films can be degraded by soil microbes and/or other factors like hydrolysis, photolysis, mechanical and thermal stress, and others and transform into natural molecules such as water, carbon dioxide, biomass, methane, or inorganic compounds [238]. Environmental degradation causes a significant loss of mechanical properties in low‐molecular‐weight biopolymers. The physicochemical properties of the materials influence the major degradation pathways for biopolymers. Surface area, molecular weight, chemical structure, crystallinity, modulus of elasticity, glass transition temperature, and melting temperature of biopolymers can be responsible for the biodegradation rate. The type of environmental processes, for example, erosion, hydrolysis, photodegradation, and thermo oxidative degradation, and factors like moisture, temperature, solar radiation, air movement, pressure, precipitation, chemicals, and micro and macroorganisms all play important roles in the efficient degradation process. Because biopolymers are long‐chain molecules, degradation may occur in different stages. Temperature, water, and sunlight first break down the long polymeric chains into monomers. Furthermore, biodegradation can take place in two ways, aerobically or anaerobically. Because anaerobic degradation produces methane gas, aerobic degradation is the preferred method for biopolymer degradation [40]. In general, hydrolysis of biopolymers involves polymeric backbone bond dissociation in the presence of water, with dissociated molecules converting to water‐soluble monomeric acids via two pathways namely bulk and surface erosion. Water molecules diffuse quickly into amorphous sites during bulk erosion, causing the biopolymer to lose strength and structural properties quickly. Surface erosion, on the other hand, causes materials to degrade first on the exterior surface, then on the interior surface [40]. Surface erosion occurs by first hydrolysing the outer surface, whereas bulk erosion hydrolyses the entire biopolymer, resulting in the hydrolytic degradation of the biopolymer's surface and its core. The degree of hydrolysis is affected by several factors, including pH, temperature, hydrophobicity, morphology, crystallinity, and porosity. In the presence of water, polyanhydrides, and aliphatic polyesters degrade in 182 days [40, 239, 240].

**FIGURE 10:**
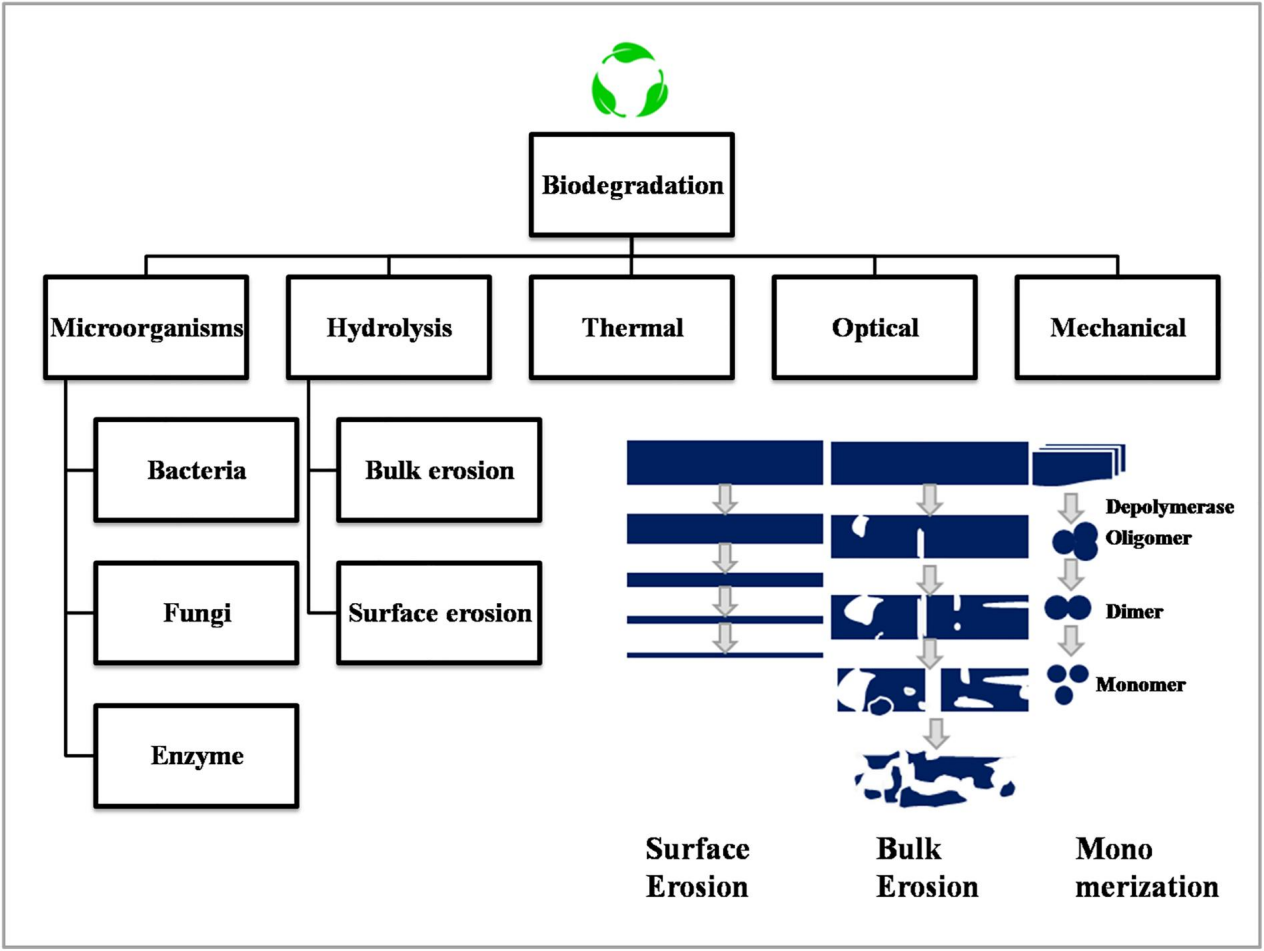
Different biopolymer degradation process [40].

To check the biodegradability of different biopolymers and biopolymer‐based composites, a soil bury‐based strategy was utilised by the scientist and further, the weight loss of the film is recorded at regular intervals to determine the degradation rate. By evaluating weight loss over time, Indumathi et al. investigated the biodegradability of a polyurethane/chitosan/ZnO nanoparticles (5wt%) film. After 28 days in the soil, the film had lost 86% of its initial weight, according to the findings, whereas the pure chitosan film degraded completely in this period [36]. It is easy to degrade biopolymers of hydrophilic nature (such as chitosan), and with high water absorption, and surface wettability. Moisture from the soil easily penetrates the polymer network, weakening it and rendering it vulnerable to hydrolysis. Larger binding forces in the nanocomposite, on the other hand, allowed the films to be retained for a longer period before entirely disassembling and degrading [166]. The addition of nanofillers often improves the biopolymer's durability. Nanofillers, on the other hand, can induce rapid deterioration in the case of inadequate dispersion, depending on the structural integrity and condition of interfacial adhesion [241]. Polyak et al. degraded amorphous Polyhydroxybutyrate films in aqueous media at various pH values (13.0, 12.5, 12.0, and 7.0), conducted over various degradation periods (7, 14, 21, and 28 days). The results showed that degradation occurs in the interior of the samples rather than on their surface because degradation does not occur randomly but rather with a greater frequency near the end of the chains. The rate of degradation increased as the pH increased [242]. Research proves that starch and starch‐based bioplastics generally possess a faster degradability rate. Pure starch degraded roughly 60% in just 10 days in an aerobic biodegradation medium. When cassava starch and yerba mate are combined, the degradation took place in only 6–12 days in the vegetal compost medium [243]. Cassava starch/chitosan/pineapple leaf fibre/ZnO film decomposed completely in 21 days in ordinary soil and 18 days in seawater. In one study, xylan‐starch‐based bioplastic proved to decompose within a month, in another 13 days after burial in the soil [244, 245]. Keratin from waste chicken feathers with ginger starch was determined that when the films were buried in soil, they degraded by more than 50% of their initial mass in 12 days [80]. In contrast, Singh et al. found rice husk‐reinforced corn starch is resistant to a higher degradability rate. Although blending biopolymers enhance their durability to some extent, that does not affect biodegradability much [68] (Figure 10).

## 17 CONCLUSION

Increasing amounts of waste and industrial by‐products pose a socio‐economic and environmental challenge. So, converting it into valuable products has become essential for achieving sustainability and economic circularity. Numerous organisations are working in this field internationally, including the Sustainable Packaging Alliance in Australia and the Sustainable Packaging Coalition in the United States, due to the importance of reducing associated environmental risks. Numerous published experimental studies have confirmed the potential of various wastes that can be good sources of biopolymers, as discussed above. Much research is still required to bring these biopolymers generated from waste to utilise at the industrial level for practical mass application. The production of biopolymers should be consistent to increase material sustainability and reduce long‐term reliance on non‐renewable resources. Furthermore, the feasibility of biopolymer products at the laboratory scale should be validated at the pilot scale before they are used in real‐world applications. Several reports confirm that blending other required materials with the biopolymer can enhance its properties and durability. At the moment, the high cost of biopolymer products is one of the major constraints to gaining competitiveness. Continuous technological advancement and innovative efforts may help reduce this cost barrier, making it a viable option for industries. Standards, certifications, and labelling are regulatory tools that can help biopolymers establish a sustainable economy. The development of such criteria is critical for the establishment of biopolymer markets, both for the generation of benefits and for overcoming uncertainties or doubts about their reliability [246]. An appropriate processing method is required to valorise each type of waste material However, experimental data and optimisation from different research can be helpful in this case. Although numerous studies are going on, it is still in its early stages of development and is currently restricted to the laboratory scale. In the near future, effective green‐based for commercial‐scale development strategies are required. National policymakers must encourage inter‐sectorial linkages to connect various enterprises and thus capitalise on waste generated by industrial and urban activities. In the nearby future, waste‐derived bioplastics will emerge as valuable materials in different fields for a wide range of cutting‐edge applications. Establishing plants to extract biopolymers from waste will benefit foreign currency reserves and job creation, in environmental sustainability manner with economic benefits. According to the research of the relevant literature, the different feedstock can affect the yield of a bio‐based production process. Furthermore, the process of sustainability and commercialisation must be well‐organised and managed. More and more research is needed to improve these value‐added materials, as well as their marketing and sustainability.

Finding packaging materials that may increase the shelf life of perishable food products are made from renewable resources, and aid the environment by reducing waste has become essential today. There is currently very little biodegradable packaging material on the market. Currently, almost 99% of today's plastics are petroleum‐based, and their continued use has been disastrous for the planet and only just 1% of the 320 million tons of plastic produced annually is made of bioplastics [247]. Due to small‐scale production and higher research and development and production costs (2–3 times higher than conventional plastics), biodegradable polymers are not as widely used in numerous industries [248]. Customers may choose not to buy a sustainable product for a variety of reasons, such as less availability, the product's higher cost, other unawareness of the issue associated with it.

However, the use of nanomaterials as fillers within the biopolymer matrix are getting popularity among scientists, due to the significant knowledge gap about their migration potential and toxicity concern, there is debate regarding their safety. Since toxicity is linked to consumer health, it is a vital area of exploration and needs to be controlled. Regulating bodies including the European Commission's Health and Consumer Protection Directorate and the United States Environmental Protection Agency are now focusing on the potential effects of nanoparticles.

## AUTHOR CONTRIBUTIONS

**Zeba Tabassum:** Investigation; Writing – original draft; Writing – review and editing. **Anand Mohan:** Conceptualisation; Resources; Writing – review and editing. **Narsimha Mamidi:** Visualisation; Writing – review and editing. **Ajit Khosla:** Data curation; Resources; Software. **Anil Kumar:** Data curation; Methodology; Project administration; Writing – review and editing. **Pratima R. Solanki:** Methodology; Resources; Visualisation; Writing – review and editing. **Tabarak Malik:** Conceptualisation; Resources; Supervision; Writing – review and editing. **Madhuri Girdhar:** Conceptualisation; Investigation; Methodology; Project administration; Supervision; Visualisation; Writing – review and editing.

## CONFLICT OF INTEREST STATEMENT

Authors have no conflict of interest to disclose.

## Data Availability

Data sharing is not applicable to this article as no new data was created or analysed in this study.
